# Characterizing RNA Oligomers Using Stochastic Titration Constant-pH Metadynamics Simulations

**DOI:** 10.1021/acs.jcim.4c02185

**Published:** 2025-03-18

**Authors:** Tomás F. D. Silva, Giovanni Bussi

**Affiliations:** Scuola Internazionale Superiore di Studi Avanzati, Trieste 34136, Italy

## Abstract

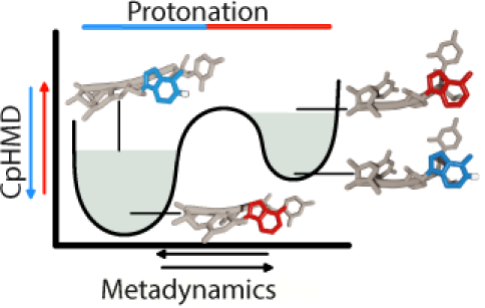

RNA molecules exhibit various biological functions intrinsically dependent on their diverse ecosystem of highly flexible structures. This flexibility arises from complex hydrogen-bonding networks defined by canonical and noncanonical base pairs that require protonation events to stabilize or perturb these interactions. Constant pH molecular dynamics (CpHMD) methods provide a reliable framework to explore the conformational and protonation spaces of dynamic structures and to perform robust calculations of pH-dependent properties, such as the p*K*_a_ of titratable sites. Despite growing biological evidence concerning pH regulation of certain motifs and its role in biotechnological applications, pH-sensitive *in silico* methods have rarely been applied to nucleic acids. This work extends the stochastic titration CpHMD method to include RNA parameters from the standard χOL3 AMBER force field. We demonstrate its capability to capture titration events of nucleotides in single-stranded RNAs. We validate the method using trimers and pentamers with a single central titratable site while integrating a well-tempered metadynamics approach into the st-CpHMD methodology (CpH-MetaD) using PLUMED. This approach enhances the convergence of the conformational landscape and enables more efficient sampling of protonation-conformation coupling. Our p*K*_a_ estimates are in agreement with experimental data, validating the method’s ability to reproduce electrostatic changes around a titratable nucleobase in single-stranded RNA. These findings provide molecular insight into intramolecular phenomena, such as nucleobase stacking and phosphate interactions, that dictate the experimentally observed p*K*_a_ shifts between different strands. Overall, this work validates both the st-CpHMD method and the metadynamics integration as reliable tools for studying biologically relevant RNA systems.

## Introduction

pH is a ubiquitous environmental factor that significantly influences the structure, chemistry, and function of various biomolecules. By influencing the protonation states of chemical moieties based on their intrinsic p*K*_a_, pH affects their overall charge and thermodynamic equilibria.^1,2^ This pH-dependent modulation is pivotal for regulating multiple biological processes, such as protein–protein interactions, nucleic acid binding, drug interactions, and conformational changes in response to shifts in the pH environment or nearby electrostatics.^3,4^ Usually, nucleic acids are not particularly sensitive to small physiological pH changes because the p*K*_a_ of nucleotides is quite far from the physiological range, and it is even further in common canonically base-paired nucleotides.^5^ However, noncanonical base pairings and diverse arrays of inter- and intramolecular interactions promote more complex and pH-sensitive electrostatic environments. For instance, some noncanonical base pairs, such as the A^+^-C wobble and G-C^+^ Hoogsteen pairs, involve protonated forms of adenine (p*K*_a_ 3.5 - N1) and cytosine (p*K*_a_ 4.2 - N3).^6^ In other instances, certain modified nucleobases, like 1-methyladenosine (p*K*_a_ 8.3 - N6) and 3-methylcytidine (p*K*_a_ 8.7 - N4), deprotonate at more basic conditions when compared to their unmodified counterparts, as they have significantly lower p*K*_a_ values closer to physiological pH (7.0–7.4).^6^ These p*K*_a_ shifts highlight that the modified nucleobases are more prone to protonation and deprotonation events, which can energetically promote or hinder pH-dependent conformational rearrangements depending on the electrostatic environment and medium pH. Although several experimental studies have explored the p*K*_a_ and related properties of nucleobases^7,8^ nucleosides^9−11^ and nucleotides,^8,12,13^ recent research has increasingly focused on the role of pH-dependent secondary and tertiary structures, such as i-motifs^14,15^ and triplexes.^16,17^ These structures are not only important for biological functions like catalysis and structural stability but also hold significant potential for biotechnological applications, including biosensors,^18−20^ drug delivery systems^21^ and molecular switches.^22,23^ Yet detailed insights into the mechanisms of action of these systems remain difficult to obtain through experimental protocols alone.

Molecular dynamics (MD) methods strongly complement experimental RNA studies by providing an atomistic description, although at shorter time scales.^24^ However, standard MD simulations typically assume fixed protonation states based on the molecule’s p*K*_a_ at physiological pH, ignoring the dynamic nature of protonation events that are modulated by the molecule’s instantaneous 3D conformation and surrounding electrostatic environment. These limitations result in an incomplete picture of biomolecular behavior, especially in complex biological systems where protonation events drive relevant conformational interactions. Constant-pH molecular dynamics (CpHMD) techniques have been developed to overcome these limitations. These methods introduce residue titration within an MD framework, thus enabling the prediction of protonation states and p*K*_a_ values of titratable groups. Importantly, they allow us to probe the dependence of the protonation state on the molecular conformation. CpHMD methods can be broadly categorized into continuous and discrete approaches. Continuous methods, typically based on λ dynamics^25^ such as PHMD^26−28^ or PME-based CpHMD^29^ or the GROMACS scalable CpHMD version,^30^ sample both conformations and fractional protonation states by extending the Hamiltonian with a pH-dependent λ particle with fictitious mass. These methods can be further divided based on whether they use implicit solvent models^26^ or explicit solvent models.^28,30−32^ All have been successfully applied to different biomolecules (e.g., nucleic acids^27,33^ and proteins),^26,34^ using various force fields (e.g., CHARMM, AMBER). Previous work on nucleic acid titration was pioneered by Honig and coworkers^35^ who performed p*K*_a_ estimations using rigid Poisson–Boltzmann calculations, and later by Pasquali and coworkers^36,37^ through constant-pH simulations on coarse-grained models, and also by Goh and coworkers^27,33^ using another CpHMD approach with all-atom models. Focusing on the all-atom dynamical model, this work examined on the continuous multisite-λ-dynamics^38^ constant pH implementation (CPHMD^*MS*λ*D*^) using the CHARMM force field. Adenosine and cytidine were used as model compounds for their p*K*_a_ calibration while obtaining good experimental agreement with their test compounds: adenosine monophosphate (AMP), cytidine monophosphate (CMP), and dinucleotide combinations (cytosine-cytosine, adenosine-adenosine, cytosine-adenosine). Discrete methods, on the other hand, usually employ a start–stop Monte Carlo (MC)/MD approach that accepts or rejects a protonation state switch for each titratable residue through a Metropolis criterion. The criterion depends on the protonation free energy of a given residue, which is calculated on a frozen conformation using an implicit solvent model, either Generalized-Born, as in PHREM^39^ or Poisson–Boltzmann (PB)^40−42^ methods. For example, the stochastic titration constant-pH method (st-CpHMD) was originally developed by Baptista et al.^40^ for proteins^43−45^ using the GROMOS force field and is now also available with CHARMM36.^46^ Within this approach, a model compound is used as a nonphysical fragment (i.e., the nucleobase) that encapsulates the p*K*_a_ of the chemical moiety within the molecule.^47^ For instance, the calibration of this p*K*_a_ (p*K*^mod^) is necessary for all nucleobases based on experimental data from nucleosides.^7^ With this procedure, the p*K*^mod^ is fine-tuned to correct for any systematic deviations from experimental p*K*_a_ caused by the PB parameters. The intricate details of the st-CpHMD method are extensively discussed in the literature.^40,43,47,48^ To the best of our knowledge, these discrete methods have never been tested on nucleic acids.

Building on the previous work and standard amino acid p*K*_a_ calibration protocol^47^ we aim to extend the stochastic titration constant pH molecular dynamics (st-CpHMD) method to nucleic acids by adapting χOL3 AMBER force field parameters. Our approach entails parametrizing the charged states of nonmodified RNA nucleobases through a restrained electrostatic potential (RESP) protocol. A first-stage calibration of individual p*K*^mod^’s using nucleoside experimental p*K*_a_ data as a reference^7^ is necessary to compute the protonation-free energies for the p*K*^mod^ model compound. Then, a second-stage validation and recalibration of the p*K*_a_ values are done with oligonucleotides of varying sizes according to available experimental data.^10−12^ This protocol integrates the effects of the phosphate backbone on p*K*_a_ shifts, analogous to amino acid p*K*^mod^ calibration with pentapeptides,^47^ resulting in p*K*_a_ values more representative of biomolecular environments. Trimer and pentamer systems of each nucleobase flanked by nontitrating residues were simulated to assess the effects on the p*K*_a_ of increasing the backbone length by measuring their Δp*K*_a_. Ideally, the method would accurately compute the reference experimental absolute p*K*_a_ values and Δp*K*_a_ shifts from the nucleoside to the pentamer system, thus describing the changes in the electrostatic environment in relation to the phosphate backbone.

**Figure 1:**
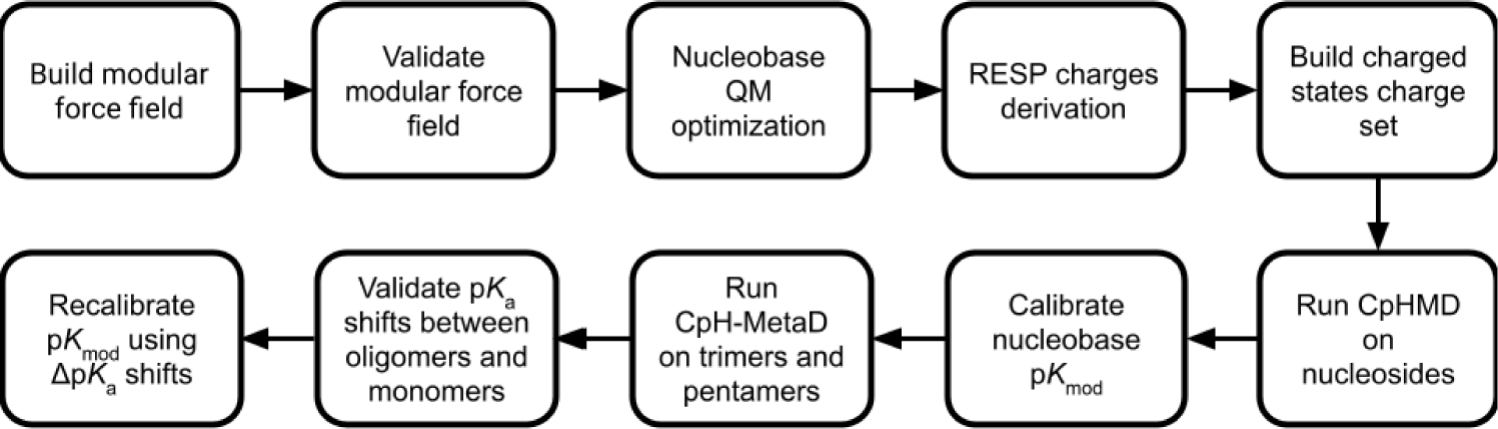
General methodological workflow of the study.

Another focus of this work is to address the challenge posed by short flexible nucleotides in MD simulations^49−51^ by integrating the CpHMD protocol with metadynamics^52^ through the PLUMED plugin. This integration focuses on improving the sampling of system-specific collective variables (CVs) without introducing a bias into the protonation space of titratable sites. Since the sites’ conformation and protonation states are intrinsically coupled, enhanced conformational sampling improves the accuracy of average protonation and p*K*_a_ predictions, particularly for well-solvated sites. The general workflow of our approach is shown in Figure 1. Our work presents a reliable and robust framework for the pH-dependent study of conformational dynamics in nucleic acids through a CpH-metaD approach.

## Methods

The st-CpHMD method by Baptista^40^ employs a start–stop sampling approach to sample the conformational space while titrating one or multiple titratable sites simultaneously between their discrete protonation states. The method uses the Poisson–Boltzmann (PB) derived free-energy terms of each titratable site to compute the likelihood of protonation states through a Monte Carlo run. Then, a short MD run is performed with the newly assigned states. In practice, the st-CpHMD method works in *N* cycles of the following workflow: 1) a PB calculation is performed on the last system conformation to report the protonation free energies of the titrating residues. These free-energies are used in an MC simulation along with the reference p*K*^mod^ to assign new integer protonation states to the titratable residues; 2) a short MD run is performed with the solute constrained to allow the water solvent molecules to adapt to the new charges of the system; 3) a longer MD run is conducted with the new protonation states to sample the conformational space. Well-tempered metadynamics is introduced in this last step using PLUMED. At the end of the run, the cycle is restarted.

### CpHMD Simulation Using AMBER χOL3 Force Field

The CpHMD extension introduced in this paper builds upon the standard AMBER parametrization^53−55^ by introducing only the charged states of the nucleotides, using the neutral states as references. Other parameters were adapted from the original force field. Parameters were derived to be compatible with the OPC water model.^56^

The new charge set parametrization used two steps of the RESP procedure^57^ on optimized geometries of the protonated and deprotonated states of all nucleobases, with riboses replaced by methyl groups as in previous work.^58^ The geometry optimization used the B3LYP functional with the 6-31G* basis set using the Gaussian16^59^ software. Then, RESP charges for each nucleobase’s neutral and charged states were derived. In this approach, we only considered a single tautomeric form for both neutral and charged states of nucleobases. Other isomers were excluded as their aqueous phase populations have been suggested to be negligible for nucleobases.^60^ Nevertheless, future work may include an additional p*K*^mod^ parametrization to account for the relative populations of these isomers, particularly considering that environmental effects might affect the relative population of the two tautomers.

In our force field, the nucleotides’ neutral states preserved the original charge set of the χOL3 force field. For the charged states, the partial charges were determined using the following protocol: 1) a QM structural optimization was performed for each neutral and charged state of all nucleobases; 2) RESP partial charges were derived for all nucleobase atoms in both neutral and charged states; 3) we calculated the charge difference between the charged and neutral states for each atom of each nucleobase; and 4) we added the calculated RESP difference for each atom to the neutral χOL3 partial charges. Thus, the charged states’ charge sets are built upon the original χOL3 parameters. Charges are shown in Tables S1–S4. Furthermore, the additional bonded and nonbonded parameters of the extra hydrogen atom necessary for both nucleotide states were based on the equivalent chemical moieties already present in the χOL3 force field parametrization. These extra hydrogens are always preserved when changing the protonation state, as we assume that the impact of protonation changes is mainly electrostatic.

For the Poisson–Boltzmann calculations, we built the DelPhi databases for the atoms’ radii and charges. In the radius determination procedure, Lennard-Jones parameters of all atom types were used, based on Lorentz–Berthelot combination rules^61,62^ to determine the radius of each atom in relation to OPC water molecules, as has been done in other similar protocols.^48^ The atomic partial charges database was built from the original χOL3 force field partial charges along with the newly derived charge set for the charged states of the nucleobases.

For technical purposes and to introduce modularity in our modified force field, the nucleobase net charge was restricted solely to the model compound (nucleobase) by rebalancing the point charges on the C1’, H1’, and N9/N1 atoms (for purines and pyrimidines, respectively). This rebalancing ensured that the nucleobase moiety had an integer charge without affecting its physical properties. These small modifications were also applied to the neutral state. To validate the modular force field, we initially performed 200 ns of MD simulations for the canonical RNA nucleosides using the original χOL3 force field. Then, new topologies were generated using the modular charge set for the canonical nucleotides, which differed in the C1’ and N1/N9 charges. Afterward, the potential energy of each system was recomputed by recomputing the energy of each trajectory using the new topologies, and then a reweighting procedure was applied to obtain reweighted observables. Finally, a comparative analysis using experimental NMR and computational ^3^J coupling data showed that the modular force field matches the original χOL3 force field, even with small tweaks in the charge set. All the mentioned parameters can be found in the following GitHub repository: https://github.com/Tomfersil/CpH-MetaD.

### Systems Setup

**Figure 2:**
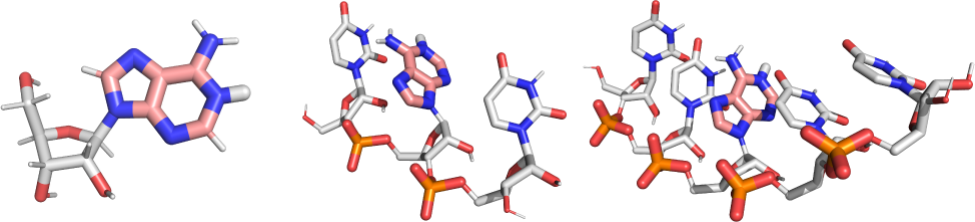
Cartoon representation of the nucleoside, trimer, and pentamer adenine systems simulated in this work. Each system exhibits different levels of phosphate backbone content: none, two, and four. For completeness, the chosen configurations represent the protonated states expected at low pH. In the single-strand systems, nontitratable nucleotides are represented with white carbon and hydrogen atoms, and the phosphate group is shown in orange and red for the phosphorus and oxygen atoms, respectively. In every system, the titratable nucleobases and protons are represented with pink carbon, blue nitrogen, and thicker white hydrogen sticks. Each system is capped with hydroxyl groups at the 3′ and 5′ ends.

**Figure 3:**
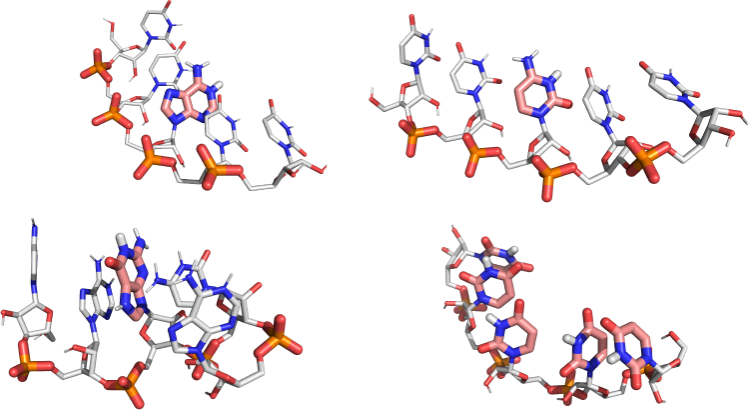
Cartoon representation of the pentamer systems simulated in this work (see Table S5). The titratable nucleobases and proton representations are described in Figure 2. Nonpolar hydrogens are not represented for improved visual clarity. The selected frames are representative of the energy basin closest to the A-form helix except for the guanine pentamer, in which a conformation was manually selected to better highlight the titrating nucleobase. The chosen configurations represent the protonated states at low pH for all systems.

Gonzalez-Olvera and colleagues studied single-stranded rU[A,C]U and rUU[A,C]UU as protonable systems^11^ and published experimental data on these molecules (Figures 2 and 3). We, therefore, use their experimental findings to validate our computational method. Since the experimental study focused on DNA single-strands, absolute p*K*_a_ values may differ from our RNA single-strands. However, the relative Δp*K*_a_ shifts between monomers (nucleosides) and oligomers (single-strands), within the same nucleic acid framework, mainly depend on the titratable nucleobase interactions with the phosphates. Therefore, they should be consistent for both RNA and DNA. Single-stranded rA**G**C, rCA**G**CA, rC**U**C and r**UUUUU** were constructed to test guanosine^10,12^ and uridine^9^ deprotonation. All systems, except for the uridine pentamer, titrate only a single central residue. For the uridine pentamer, we allow all uridines to titrate simultaneously.

Each system was built using PyMOL^63^ with the neutral protonation states. All systems were placed in a solvated rhombic dodecahedron box. As highlighted by Sequeira et al.^46^ an important consideration when adapting force fields such as AMBER or CHARMM to the st-CpHMD method is the treatment of long-range electrostatics with particle mesh-Ewald (PME) and the charge variation associated with titration events. Hence, each system’s net charge was neutralized using the appropriate number of counterions for the neutral state of the oligomer and the experimental ionic strength. PME background correction was used to compensate for the charge variations due to titration in these systems. We note that alternative approaches modify the charge of a single water molecule to reach neutrality.

### p*K*^mod^ Calibration

The choice of a model compound is an important step in the st-CpHMD method. A model compound is defined as a reference fragment of the titrating molecule, which in this work refers to the titrating nucleobase. The p*K*^mod^ is the p*K*_a_ of the model compound that defines the protonation probability without considering interactions with other surrounding chemical moieties. In practice, the p*K*^mod^ is an internal reference value within the methodology that centers the titration curve of the titrating model compound. Changing the p*K*^mod^ is equivalent to shifting the titration curve along the pH axis. Therefore, a simulation performed at a given pH *X* is equivalent to performing it at pH *X* + *Y* if the initial p*K*^mod^ guess is shifted by *Y*. The p*K*^mod^ calibration requires an initial guess to be assigned, after which a set of st-CpHMD simulations is performed to compare the calculated value with the experimental p*K*_a_ data. Ideally, the p*K*^mod^ would be equal to the experimental nucleobase p*K*_a_. However, this calibration procedure is required to correct systematic deviations of the Poisson–Boltzmann energy estimations.

Protonation and deprotonation events occur at the nucleobase, at N1 or N3, for purines and pyrimidines, respectively. Due to limited long-range electronic effects, we restricted the charge variation of (de)protonation of the titratable site (±1) within the defined model compound. Hence, the point charges of the sugar and phosphate groups remain unchanged regardless of the protonation state. Under this assumption, our modified force field is constructed using a modular-based rationale, where the phosphate group, the ribose, the hydroxyl group cap (3′ and 5′), and each nucleobase are defined as unique residues. The major advantage of this modular approach is that future inclusion of modified nucleobases in the force field will only require defining the nucleobases and their respective protonation states by following this work’s protocol.

In this work, we calibrated the model compounds p*K* (p*K*^mod^) by applying a similar rationale to that used in the calibration of titratable amino acid residues^45−47^ though with an additional initial step. In the first step, we assigned an initial p*K*^mod^ value and ran an iterative procedure of short 3 × 100 ns CpHMD simulations (adenine) or 1 × 100 ns CpH-MetaD simulations of all nucleosides under equivalent experimental conditions (300 K and 0.1 M). The CVs biased in the metadynamics simulations were the χ glycosidic angle to promote *syn*/*anti* transitions and a sugar puckering variable to promote transitions between the C2′ and C3′ endo states.^64^ A single 100 ns CpH-MetaD run exhibited faster conformational convergence compared to the 3 × 100 nsCpHMD runs (see Figure S1). After obtaining the titration curve, we calculated the p*K*_a_ from this first iteration of simulations. If the estimated p*K*_a_ deviated from the reference experimental p*K*_a_ of the nucleoside, we corrected the initial p*K*^mod^ by shifting it by the same magnitude in the opposite direction. Multiple iterations were performed until a final p*K*^mod^ that reproduces the experimental p*K*_a_ data was obtained. The resulting shifts were smaller than 0.15 pH units, indicating that the electrostatic potentials derived from the Poisson–Boltzmann calculations properly describe the titratable site and the surrounding environment in the nucleobase systems.

The second p*K*^mod^ calibration step took place after the oligonucleotide CpH-metaD simulations (see Table S5 for all oligonucleotide sequences and simulation parameters). As referenced in the Introduction section and further discussed in the Results section, the phosphate backbone modulates the protonation behavior of the nucleobases in a more complex biomolecular environment. Similarly to amino acid calibration protocols^47^ we applied *a posteriori* corrections to the final p*K*^mod^ values to accurately recover the experimental absolute p*K*_a_ values of the single-strands by using the p*K*_a_ shifts referenced in experimental data. Thus, these p*K*_a_-shift-based corrections adjust the absolute p*K*_a_ predictions to improve the method’s ability to describe changes in the biomolecular environment. This correction was applied by simply shifting the p*K*^mod^ by the Δp*K*_a_ between the experimental p*K*_a_ reference and the model trimer p*K*_a_. For the protonable systems, the reference experimental p*K*_a_ data were obtained by Gonzalez-Olvera^11^ on nucleotides, trinucleotides, and pentanucleotides DNA constructs. Due to the lack of experimental data on similar single-strand RNA, we used these constructs for validation. For the guanine systems, the final calibration used the data obtained by Acharya et al.^12^ for RNA constructs, and the uridine systems were compared to the RNA experiments conducted by Izatt et al.^7^

### MM/MD and CpHMD Settings

CpHMD and CpH-MetaD simulations were run using version 2022.3 of the GROMACS package^65,66^ and the open-source, community-developed PLUMED library^67^ version 2.8.1.^68^ All simulations used the previously described modified χOL3 AMBER force field^55^ with the OPC water model.^56^

A Verlet 1.0 nm cutoff scheme was applied for the PME treatment of nonbonded interactions. van der Waals interactions were truncated at 10 Å. The integrator time step was 2 fs and the conformations were sampled from an NPT ensemble. Unless otherwise specified, the temperature coupling scheme used was the v-rescale^69^ at 300 K with a relaxation time of 0.1 ps, coupled to the solute and solvent separately. The system pressure was kept constant with a c-rescale barostat^70^ at 1 bar, with a relaxation time of 2 ps and a compressibility of 4.5 × 10^–5^ bar^–1^.

### Poisson–Boltzmann/Monte Carlo Simulations

The DelPhi V5.1 program^71^ was used to perform Poisson–Boltzmann calculations. The solute molecular surface was defined by a 1.4 Å radius probe, an ion-exclusion layer of 2.0 Å, and an ionic strength of 0.1 or 0.01 M depending on the experimental conditions of each system (see Table S5). The dielectric constants used were 2 and 80, for the solute and solvent, respectively. A two-step focusing procedure was conducted for electrostatic potential calculations by defining two grids of 91 vertices. The coarse grid had a 1 Å spacing between the grid points, while the smaller grid had 0.25 Å. The defined relaxation parameters were 0.20 and 0.75, for linear and nonlinear interaction, respectively. Background interaction calculations were not truncated and the electrostatic potential convergence threshold was 0.01 kT/e.^72−74^

The PETIT program performed the MC calculations of the residues’ protonation states, using the free energy terms obtained from the PB calculations.^75^ For each conformation, 10^5^ MC cycles were performed, and each cycle corresponded to a trial change of each site and of pairs of sites with an interaction larger than 2 p*K*_a_ units.

### Metadynamics Integration and Settings

In this work, we integrated the well-tempered metadynamics algorithm within the MD production phase of the CpHMD cycle. In well-tempered metadynamics, the system is biased by a smoothly converging history-dependent potential^76^ along a chosen collective variable (CV).

During the MD phase of each cycle, metadynamics was restarted from the bias potential generated in the previous cycle and new Gaussian potentials were deposited. Restarts were performed by reading the potential from a grid. The final conformation was saved to be used in the PB/MC calculation.

In the oligomer simulations, we ran single 1.5 and 3 μs CpH-MetaD simulations for the trimer and pentamer systems. The CVs biased in the metadynamics simulations were the χ glycosidic angle and the eRMSD.^77,78^ The glycosidic torsion promotes transitions between phosphate-exposed (*syn*) and phosphate-shielded (*anti*) states. The eRMSD measures the relative arrangement between nucleobases in a molecule with respect to a reference fully stacked A-form conformation. Thus, it is a quantitative measure of base-stacking interactions in single-strand RNA molecules. Each system’s pH range and number of simulations were specifically chosen to interpolate their titration curve and p*K*_a_. Specific details of each system can be found in Table S5.

### Analyses and Error Calculations

#### ^3^J Scalar Couplings in Nucleosides

The Karplus equations back-calculate the ^3^J scalar couplings from the torsional angles. Experimental reference data^79^ were used to validate the ensembles for each nucleoside. The parameters for the Karplus equations for χ and χ′ torsional angles were obtained from Munzarová et al.^80^ while the sugar parameters were obtained by Condon et al.^81^

#### Structural Characterization of the RNA Oligonucleotides

We characterized the oligonucleotides’ structure using the chosen collective variables: the eRMSD, a nucleic acid-specific measurement that evaluates the nucleobases’ orientation and their relative positions, and the glycosidic χ angle. The analyses were performed using the PLUMED software and then reweighted to obtain unbiased populations.^82^

#### p*K*_a_ Calculations and Electrostatic Contributions

We estimated the p*K*_a_ values by obtaining a titration curve for each system and then taking the midpoint of the titration. The titration curve was obtained by fitting the average protonation states of each CpHMD simulation to the Henderson–Hasselbalch (HH) equation (eq 1):
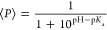
1where ⟨*P*⟩ is the average protonation, pH is the assigned simulation pH, and p*K*_a_ is the fitted parameter. For the CpH-metaD simulations, the average protonation was obtained using the reweighting procedure introduced in^82^
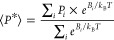
2where ⟨P*⟩ is the weighted average protonation for a given CpH-MetaD simulation and *B*_*i*_ is the bias accumulated at the end of the metadynamics simulations calculated on the coordinates corresponding to the *i*-th frame. These weighted average protonations were fitted to the HH equation.

In a different approach, we used binless WHAM^83−85^ to derive pH-dependent properties such as the p*K*_a_, pH scans of the 2D energy maps, and the average protonations of the identified energy minima. The procedure consisted of concatenating all CpH-MetaD equilibrated trajectories of a given system and then recomputing the biases for each of the simulations’ accumulated bias potentials using the concatenated trajectory. Afterward, we corrected each bias with a protonation-dependent contribution for each frame:
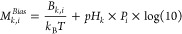
3thus obtaining a bias matrix containing the biases for each reweighted bias potential (*k*) of the concatenated trajectory, which depends on each frame’s protonation state (*P*_*i*_) and the simulation pH (*pH*_*k*_). Then, we computed the weights using this bias matrix and the protonation states of the concatenated trajectory, using a binless WHAM implementation https://bussilab.github.io/doc-py-bussilab/bussilab/wham.html. After applying this procedure, we were able to compute weights at arbitrary pH values to reweight any observable property, such as 2D energy maps, p*K*_a_, and average protonations.

The p*K*_a_ error values were calculated using a bootstrap approach. For the HH fits, we partitioned each protonation time series into 10 equally sized blocks and then performed bootstrap on the blocks with 500 iterations. We performed a new HH fit for each resample, thus calculating a new p*K*_a_. The error was obtained from the standard deviation of the resampled p*K*_a_ histogram. For the WHAM p*K*_a_ values, we partitioned the protonations and biases of each pH simulation into 4 blocks and then performed bootstrap sampling (500 iterations). Each resample generated new weights, which were then used to calculate new p*K*_a_ values. The final error was calculated as previously.

Analysis of time series of observables was performed using the GROMACS package. Further analyses were performed using in-house Python scripts or previously specified modules. Block analysis was conducted for each observable and then the error was obtained through the bootstrap procedure (1000 iterations).

## Results

In this work, we parametrized charges for protonated and deprotonated nucleotides that are relevant within the pH range 3.0–5.0 and 9.0–11.0, respectively, calibrated their p*K*^mod^ using their respective aqueous p*K*_a_’s, and tested them in several oligomers for which experimental data are available. One key factor affecting the nucleobases’ p*K*_a_’s is the electrostatic effect of the phosphate backbone, which stabilizes positively charged states (adenine, cytidine) and destabilizes negatively charged states (guanosine, uridine). Single-stranded oligonucleotides, possessing multiple phosphate groups, are ideal for validating our method’s ability to capture these backbone-dependent protonation variations and to reproduce experimental p*K*_a_ values accurately. Each titratable nucleotide was simulated within trimer and pentamer systems to capture the phosphate-dependent electrostatics effect on the nucleobase (Δp*K*_a_). Moreover, the chosen flanking residues were nontitratable within the pH range of interest (except in the uridine pentamer system), to prevent protonation coupling effects with other titratable residues. Simulations were performed using a combination of constant-pH molecular dynamics and metadynamics. Nucleobase protonation is influenced by solvent exposure and electrostatic interactions and our collective variable (CV) choices enhance the sampling of these interactions. Specifically, the eRMSD improves base stacking/unstacking, affecting solvent exposure of the titrating nucleobase, while the glycosidic angle enhances the sampling of the distance between the titrating nucleobase and the electrostatically negative phosphate backbone. In the following sections, we report the results of the MD simulations for the adenine and uridine systems. Results for other systems are presented in the Supporting Information.

### Testing Force Field Modularity

As mentioned in the Methods section, we redesigned the force field by partitioning each nucleotide chemical moiety (phosphate, sugar, and nucleobase) into a separate residue. This force field modularity aims to facilitate the future inclusion of modified nucleobases. However, the procedure required a small charge rebalance around the C1′, H1′, and N9/N1 atoms (purines/pyrimidines, respectively). The root-mean-square error of ^3^*J* scalar couplings relative to experimental nuclear magnetic resonance data (in Tables S6–S9) shows that the modular charge set is comparable to the original force field when assessing the deviation from experiment. Furthermore, the populations of *syn* and *anti* states of the glycosidic bond angle are very close to those obtained with the original charge set (Table S10). These results indicate that enforcing modularity did not affect the overall force-field quality.

### Protonable Nucleobases—Adenine and Cytosine

**Figure 4:**
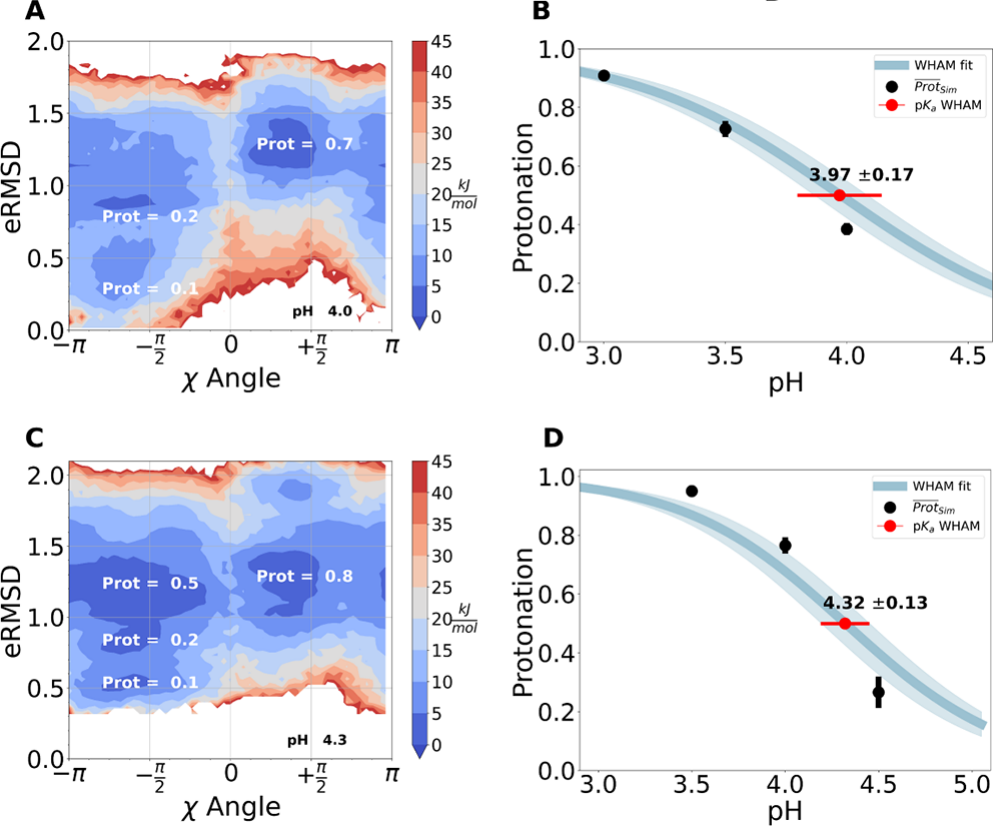
CpH-MetaD simulations estimate the p*K*_a_ shifts in the adenine systems. Panels (A,C) depict 2D free-energy maps as a function of the χ angle of the titratable adenine and the rupture of stacking interactions (eRMSD) for the rU**A**U and rUU**A**UU, respectively. The major energy minima are labeled with their average protonation (i.e., population of protonated conformations), as “Prot” at pH = 4.0 and 4.3, respectively. Panels (B,D) report the titration curves (gray lines) of the rU**A**U and rUU**A**UU, respectively. The data were obtained through a binless WHAM reweighting procedure using equilibrated data from all CpH-MetaD simulations. The corresponding p*K*_a_ values are indicated by red circles. Average protonation and respective errors from individual simulations are plotted as black circles. Standard errors were estimated using the bootstrap method.

After performing the p*K*^mod^ calibration (see the p*K*^mod^ calibration section of Methods), we conducted CpH-metaD simulations to validate the p*K*_a_ estimations of the protonable nucleobases, adenine (A—p*K*_a_ 3.5) and cytosine (C—p*K*_a_ 4.2), within more realistic biomolecular environments. The analysis was performed on equivalent trimer and pentamer systems—rU**A**U/rUU**A**UU (Figure 4) and rU**C**U/rUU**C**UU (Figure S2).

In Figure 4A, the 2D free-energy map highlights the energy minima of the rU**A**U system at pH 4.0 (close to the measured p*K*_a_) as a function of the eRMSD from the A-helix and the χ angle of the titratable nucleobase. Specifically, the eRMSD CV is anticorrelated with nucleobase stacking, while the χ angle is correlated with the proximity of the titratable site to the phosphate backbone. The map displays two distinct energy minima in the *anti* region of the titratable nucleobase at low (eRMSD < 0.7) and medium (0.7< eRMSD < 1.0) eRMSD values, and a single major energy minimum for the *syn* states  at high eRMSD values (1.0 < eRMSD < 1.5). Visual analysis of the representative structures for each minimum shows progressive unstacking from fully stacked at low eRMSD regions to fully unstacked at high eRMSD regions (Figure S4). This interpretation applies to all systems, as evidenced by the representative structures and the 2D maps in the Supporting Information. Average protonation values estimated for each energy minimum reveal a high dependency on conformation. As stacking interactions are progressively lost (Figure S4), the average protonation increases, with the highest protonation value usually corresponding to the *syn*/unstacked conformation due to closer interactions with the phosphate groups. Indeed, progressively increasing the pH leads to more energetically favorable *anti* conformations, while also penalizing the higher protonation *syn* states (Figure S11), as evidenced by the changes in the energy minima. This phenomenon is further supported by the relative p*K*_a_ shifts observed in stack-specific p*K*_a_ calculations (Figure S23). Due to increased solvent and phosphate backbone exposure, unstacked conformations exhibit a p*K*_a_ upshift relative to the global titration curve. In contrast, stacked conformations exhibit a downshifted p*K*_a_ since the desolvation effect and reduced phosphate exposure hinder protonation events in these conformations compared to partially and fully stacked states at the same pH range (Figure S23). Furthermore, these results suggest that the p*K*_a_ values of all studied systems are mainly determined by the partially stacked structures, as evidenced by the overlapping titration curves. Similar protonation-dependent behavior was observed for the rU**C**U system, as shown in Figures S6, S13, and S23.

The longer sequence in the rUU**A**UU system significantly changes the CV space and the protonation behavior of the titratable adenine. The 2D free-energy map at pH 4.3, close to the measured p*K*_a_ value, displays three minima in the *anti* region of the titratable nucleobase and a single energy minimum for the *syn* state (Figure 4). Similarly to the trimer system, average protonations remain anticorrelated with conformational stacking (Figure S5), as evidenced by the high average protonation (+0.8) observed for *syn* unstacked states relative to stacked *anti* states. Progressively more basic pH environments energetically favor the stacked conformations (lower eRMSD states), as seen in Figure S16. This increase in thermodynamic stability is also observed consistently in the pentamer system and the adenine trimer systems.

Concerning the p*K*_a_ estimations, our predictions for the protonable trimers were as follows: 3.97 ± 0.18 for rU**A**U (Figure 4B) and 4.57 ± 0.13 for rU**C**U (Figure S2B), see also Table S11. Both trimers’ p*K*_a_’s are similarly upshifted relative to their respective monomer values resulting in a theoretical Δp*K*_a_ (0.35/0.32 in Table 1) that is larger than the experimental Δp*K*_a_ (0/0.1 for rU**A**U/rU**C**U obtained from the Gonzalez-Olvera data).^11^ Thus, our model did not accurately reproduce the electrostatic impact of changing from a monomer to a trimer. Interestingly, our model properly captures the electrostatic changes in the titrating residue environment when going from a trimer to a pentamer. The pentamers’ measured p*K*_a_’s are 4.32 ± 0.14 for rUU**A**UU and 5.07 ± 0.18 for rUU**C**UU (see also Table S11). The resulting theoretical Δp*K*_a_’s between pentanucleotides and trinucleotides, + 0.35 and +0.50 for the rUU**A**UU and rUU**C**UU, are compatible with the experimental Δp*K*_a_^*exp*^ (+0.38 and +0.55 in Table 1, respectively) for both systems, despite overestimating their absolute p*K*_a_ values.^11^ For the rUU**C**UU system, we used the Henderson–Hasselbalch (HH) fit p*K*_a_ as a reference due to discrepancies in the WHAM p*K*_a_ value, which are discussed in depth in the Discussion section.

**Table 1:** Table of Δp*K*_a_ Values for the Protonable (Adenine, Cytosine) Titratable Sites (in Bold) of Single-Stranded Oligomer Systems Obtained before the Final Calibration Step^a^

Oligomers	Reference Δp*K*_a_	Predicted Δp*K*_a_
A: Δp*K*_a*T*-*M*_	+0.00	+0.35
A: Δp*K*_a*P*-*T*_	+0.35	+0.38
C: Δp*K*_a*T*-*M*_	+0.10	+0.32
C: Δp*K*_a*P*-*T*_	+0.50	+0.55

a The columns refer to the reference experimental Δp*K*_a_ values under different experimental conditions from González-Olvera^11^ and the predicted Δp*K*_a_ values from the CpH-MetaD simulations. Δp*K*_a_ with the T-M label refer to the p*K*_a_ difference between trimer and monomer, while the P-T label refer to the pentamer and trimer.

Our method aims to reproduce both the relative shifts and absolute p*K*_a_ values of titrating nucleobases in biomolecular environments with varying-sized phosphate backbones. Our calculations did not reproduce the absolute p*K*_a_ values (see Table S11) nor the correct p*K*_a_ shift between the nucleoside and the trinucleotide (see Table 1). However, it predicted the proper p*K*_a_ shift between a trimer and a pentamer. Therefore, we recalibrated our p*K*^mod^ so the method reproduces the absolute p*K*_a_ value of the trimers and pentamers, preserving their relative p*K*_a_ shift while excluding the nucleoside. We recalibrated the p*K*^mod^ using the absolute experimental p*K*_a_ values for trimers. As the trimers’ reference p*K*_a_ values are based on DNA constructs, an additional correction was included to account for the differences between RNA and DNA systems. In addition, we had to account for the experimental discrepancy reported in the reference work by González-Olvera et al.^11^ relative to previous literature.

Therefore, the adenine p*K*^mod^ was corrected by −0.42 pH units, while the cytosine p*K*^mod^ was shifted by −0.2. These values are defined as Δp*K*_a_ values measured between a hypothetical RNA trimer (based on the González-Olvera work) and our theoretical model (see Table S12).

In sum, our model can accurately describe the electrostatics and protonation variations across protonable trinucleotides and pentanucleotides, whereas it overestimated the shift from nucleotide to trinucleotide. Hence, we recommend using the final recalibrated p*K*^mod^ for larger constructs. With this parametrization, the model is not expected to reproduce the experimental p*K*_a_ for a single nucleoside in water.

### Deprotonable Nucleobases—Guanosine and Uridine

**Figure 5:**
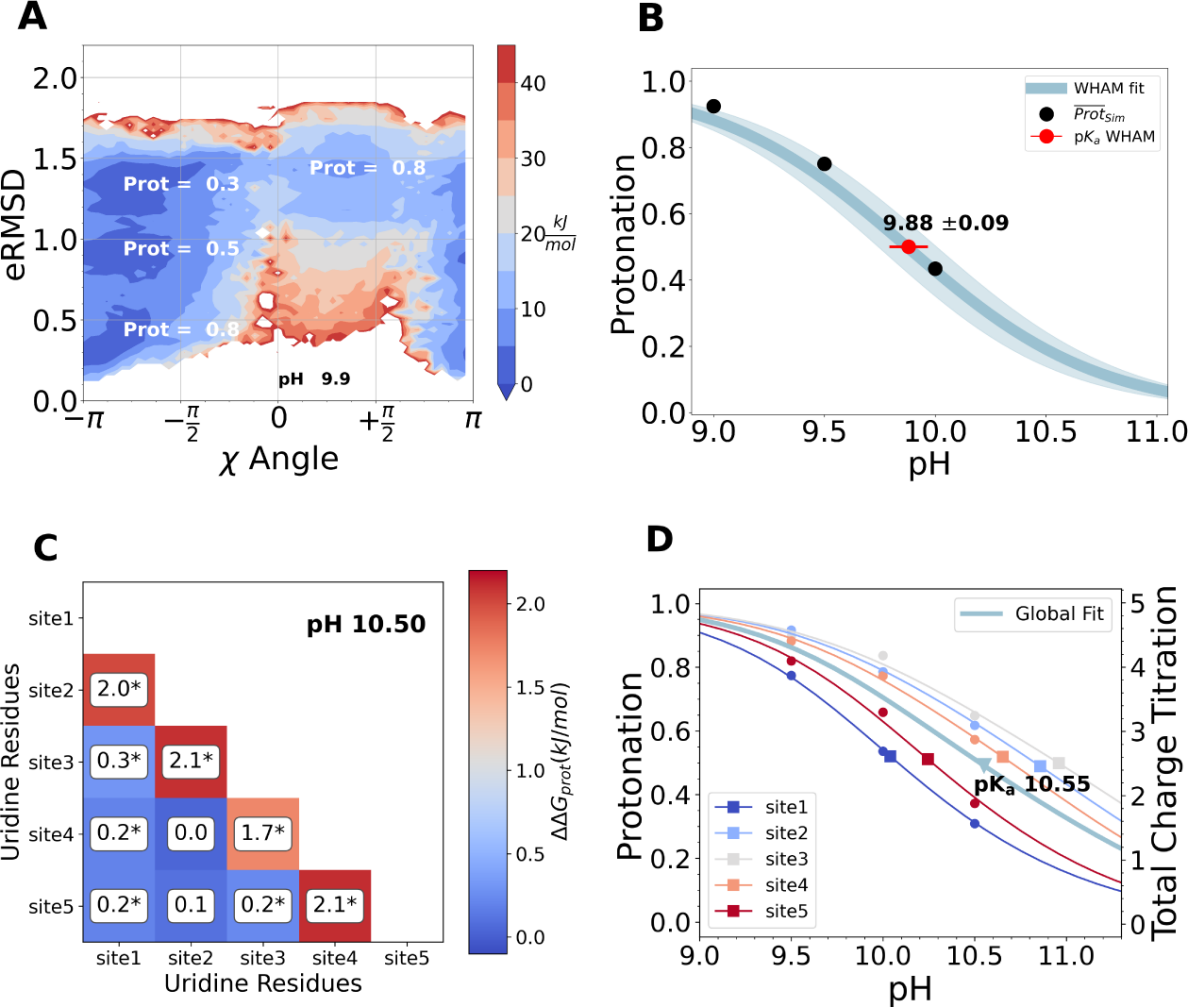
CpH-MetaD simulations estimate the p*K*_a_ shifts in uridine systems. Panel (A) depicts 2D free-energy maps as a function of the rupture of stacking interactions (eRMSD) and the χ angle of the titratable uridine in rC**U**C. The major energy minima are labeled with their average protonation (i.e., population of protonated conformations), as “Prot” at pH = 9.9. Panels (B,D) report the single and total titration curves (gray lines) of rC**U**C and r**UUUUU**, respectively, obtained through a binless WHAM reweighting procedure. For rC**U**C, the p*K*_a_ value is marked by a red circle, while the individual p*K*_a_ values of r**UUUUU** are labeled by colored squares. These p*K*_a_ values are compiled in Table S13. In panel (D), the error bars are not displayed for visual clarity. Standard errors were estimated using a bootstrap method. Panel (C) displays a pairwise protonation cooperativity matrix at pH = 10.0 (other matrices are available in the Supporting Information). The ΔΔ*G* values indicate the likelihood of simultaneous protonation of two sites in a multititratable strand. After a bootstrapping procedure, statistical significance (*) was calculated using the Benjamini–Hochberg method.^86^

For the deprotonable nucleobases, uridine (U—p*K*_a_ 9.2) and guanine (G—p*K*_a_ 9.2), the same calibration protocol was applied using different single-strand RNA sequences: rA**G**C and rCA**G**CA (Figure S3) for guanine^10,12^ and rC**U**C and r**UUUUU** (Figure 5) for uridine.^9^

In the rC**U**C system, the three identified minima across the *anti* states region (—Figure 5A) denote higher average protonation for more stacked conformations (lower eRMSD). This suggests that nucleobase stacking effects correlate with protonated states, which is further supported by the progressive p*K*_a_ upshift of increasingly stacked states (Figure S23). In contrast, unstacked *syn* states  are, on average, more protonated and less energetically favorable than the other populations. These conformations are more globular and disordered (Figure S8), tending to increase the solvent exposure of the titratable group while reducing phosphate backbone interactions. In these electrostatic environments, deprotonation events are facilitated (Figure S22), and a p*K*_a_ downshift is observed (Figure S23). Partially stacked conformations (0.8 < eRMSD < 1.0) and fully stacked conformations (eRMSD < 0.8) (Figure S8) stabilize the protonated state at any pH value, as shown by the typically higher average protonation values in Figure S22. Fully unstacked conformations exhibit distinct behavior depending on the χ angle of the titratable site. As a pyrimidine, the *syn* conformations in a trinucleotide are less likely to occur (i.e., less energetically favorable) due to nucleobase steric hindrance, which is also observed at high pH values. When they occur, *syn* states are on average more protonated than their *anti* counterparts due to the proximity of the titratable N3 to the negatively charged phosphate backbone. Concerning the rA**G**C system, stacking effects appear more relevant to the thermodynamic stability of the different conformational states than in the other studied systems. Partially stacked (0.8 < eRMSD < 1.0) and alternatively stacked (eRMSD ≈ 1.7) conformations are more thermodynamically stable than other conformations regardless of the medium pH and average protonation (Figure S18). In particular, the alternative stacked conformation (Figure S9) exposes the nucleobase to the solvent while stacking with the purine ring of the flanking adenosine. These effects strongly stabilize the protonated state in this ensemble, even under highly basic conditions.

While the flanking uridines conferred less stable stacked structures for the previously discussed systems, the rCA**G**CA pentanucleotide exhibited a lower likelihood of unstacked conformations. The two identifiable energy minima in the *anti* states region span from the fully to the partially stacked states (Figures S10 and S20). These results suggest that unstacked and partially stacked conformations favor deprotonation events due to greater solvent exposure than fully stacked conformations, as supported by the p*K*_a_ downshift of unstacked and partial conformations (Figure S23). Meanwhile, in the *syn* region, the proximity to the phosphate backbone increasingly disfavors stacked conformations in more basic media (Figure S20). As seen in the trinucleotide, the thermodynamic stability of rCA**G**CA conformations with distinct degrees of stacking interactions is less pH sensitive than the other previously discussed systems.

In the deprotonable trimer systems, the theoretical p*K*_a_ estimations slightly deviated from the experimental values for the uridine system—p*K*_a_ 9.88 ± 0.09 (p*K*_a_^*exp*^ 9.5)—and for the guanosine system—p*K*_a_ 10.25 ± 0.14 (p*K*_a_^*exp*^ 10.1) (see Table S11). The theoretical Δp*K*_a_ measured between the trimer and the nucleoside—+ 0.6 and +1.2—was close to the experimental Δp*K*_a_^*exp*^ (see Table 2): + 0.3^9^ and +0.9^12^ for rC**U**C and rA**G**C, respectively.

**Table 2:** Table of Δp*K*_a_ Values for the Deprotonable (Guanosine, Uridine) Titratable Sites (in Bold) of Single-Stranded Oligomer Systems Obtained before the Final Calibration Step^a^

Oligomers	Reference Δp*K*_a_	Predicted Δp*K*_a_
G: Δp*K*_a*T*-*M*_	+0.90	+1.20
G: Δp*K*_a*P*-*T*_	+0.50	+0.52
U: Δp*K*_a*T*-*M*_	+0.30	+0.60
U: Δp*K*_a*P*-*T*_	+0.50	+0.65

a The first column presents the reference experimental Δp*K*_a_ values under different conditions for uridine (measured by Clauwaert and coworkers^9^) and guanosine (measured by Acharya^12^). The second column presents the predicted Δp*K*_a_ values from the CpH-MetaD simulations. Δp*K*_a_ values with the T-M label refer to the p*K*_a_ difference between the trimer and monomer, while the P-T label refers to the difference between the pentamer and trimer.

Concerning the rCA**G**CA system, our p*K*_a_ prediction of 10.77 ± 0.23 is close to the experimental p*K*_a_ of 10.6 (Figure S3). Similarly to rUU**C**UU, the WHAM p*K*_a_ calculation was significantly underestimated relative to the experimental value and the HH fit (Table S11); therefore, we used the HH fit as a reference. Still, the Δp*K*_a_ (+0.52) between the rCA**G**CA pentanucleotide and the rA**G**C trinucleotide agreed with their experimental Δp*K*_a_^*exp*^ (+0.50) as shown in Table 2.

The r**UUUUU** system presented a distinct challenge compared to the other systems due to the increased complexity introduced by simultaneous multisite titration and site–site coupling effects. The macroscopic p*K* estimation (10.55 ± 0.2) was extrapolated from fitting the individual charges of the five titratable sites to the Henderson–Hasselbalch (HH) equation. The predicted value was upshifted (+0.55) relative to the experimental p*K*_a_^*exp*^ (10.0). Since the experimentally measured titration quantities do not depend directly on RNA structure, we interpreted our results using the same assumption as in the experimental work, namely that the midpoint of charge titration corresponds to the macroscopic p*K*_a_ (Table S11). Still, the Δp*K*_a_ between rC**U**C and r**UUUUU** corroborated well with the experimental value (see Table 2). We also computed each nucleotide’s individual p*K*_a_ values (Table S13) and pairwise cooperativity protonation free energy. These ΔΔ*G* values indicate the probability of simultaneous protonation/deprotonation events relative to single-site titration. Figures 5C,D and S24 highlight that the topological position impacts the simultaneous protonation probability and, consequently, the individual p*K*_a_s. Terminal residues are more strongly coupled to their neighbor relative to more distant nucleotides at all tested pH conditions. The deprotonation of central nucleotides becomes more hindered by the titration of multiple neighbors, reflected in an increased upshifted p*K*_a_, particularly for the third titration site, as shown for the central uridines in Figure 5D.

Similarly to the previous systems, our calculations did not accurately reproduce the Δp*K*_a_ shift from nucleoside to trinucleotide, and an equivalent recalibration procedure was applied. However, for the deprotonable systems, the simulated systems are directly comparable to the experimental RNA data. Thus, the p*K*^mod^ correction is simply the Δp*K*_a_ between the experimental p*K*_a_ reference and the model trimer p*K*_a_. The guanosine p*K*^mod^ was corrected by −0.15, while the uridine p*K*^mod^ was shifted by −0.38 (see Table S12).

As in the protonable systems, the method accurately predicts the p*K*_a_ variations between the trimer and pentamer deprotonable systems, while failing to estimate the electrostatic changes from a nucleoside. Nonetheless, this is within the model’s expectations, as the main purpose is to reproduce complex electrostatic environments within phosphate backbones. Remarkably, the method can estimate individual p*K*_a_ values of coupled titratable sites and the macroscopic titration midpoint with good agreement with experimental data.

## Discussion

In this work, we initially developed charge set parameters for protonated and deprotonated nucleotides suitable for constant-pH MD simulations of RNA, using the χOL3 force field as a template—one of the most commonly adopted parametrizations. Our parametrization follows a modular approach that is compatible with the existing force field, thereby simplifying the future parametrization of modified nucleobases in both the force field and DelPhi databases. For the DelPhi databases’ parametrization for the Poisson–Boltzmann calculations, the reported parameters follow previously established protocols for standard st-CpHMD methodology and can be further optimized in future iterations.

Additionally, we integrated well-tempered metadynamics within the st-CpHMD method to facilitate the simultaneous sampling of conformational and protonation states. Enhanced sampling is crucial for the accurate calibration of nucleic acid parameters, as even short single-strand oligomers require extensive sampling to sufficiently explore their conformational space. For the investigated systems, using computing nodes with 1 GPU and 4 cores, we achieved a simulation speed of approximately 130 ns/day. Approximately 25% of the computational cost arises from PB calculations, 25% from solvent relaxation, and 50% from MD simulations. Several environmental factors influence nucleobase titration, including other titrating chemical moieties, charged neighbors, pH, solvation, and structural effects. These factors alter the free-energy landscape and, consequently, the likelihood of conformational rearrangements or binding events with other molecules, such as ions, ligands, or proteins. The st-CpHMD method balances these diverse contributions, offering an accurate and robust description of RNA molecules by complementing structural dynamics with protonation events in pH-dependent environments. In practice, the medium pH modulates the sampling probabilities of a given conformation by shifting the thermodynamic equilibrium between energy minima driven by charge variations in the residues. As a result of this protonation-conformation coupling, protonation space sampling can be indirectly improved by enhancing conformational sampling in a given collective-variable (CV) space at a user-defined pH. The observed differences in the average protonation values of distinct energy minima result from the individual microscopic p*K*_a_ values of each conformational ensemble. These differences are related to the pH dependence of the conformation-specific thermodynamic stabilities.^87,88^ Hence, the experimentally observed macroscopic p*K*_a_ is a weighted average of conformation-specific microscopic p*K*_a_ values.

For this work, the nucleobase p*K*^mod^ validation required single-strand oligomer constructs to mimic typical complex electrostatic environments of RNA biomolecules. Because limited conformational sampling could reduce p*K*_a_ prediction accuracy and hinder the validation against experimental data, introducing well-tempered metadynamics within the CpHMD method (CpH-MetaD) was pivotal in our calibration procedure, promoting convergence in the simulated RNA oligomers. In the future, this enhanced sampling method can complement other methods, such as pH replica exchange.^39,89,90^

For the studied systems, the CpH-metaD approach successfully sampled their conformational landscape and protonation space, thereby validating the applicability of the method and providing further atomistic insight into each system. Consequently, the results support that this approach provides robust and accurate absolute p*K*_a_ estimations and p*K*_a_ shifts when using a binless WHAM procedure across distinct single-strand RNA molecules and pH values. Nonetheless, some systems, such as the rUU**C**UU and rCA**G**CA pentamers, remained challenging to converge, leading to inconsistencies in the binless WHAM procedure and resulting in underestimated p*K*_a_ values relative to the experimental and HH-fit data (Table S11). Future work may involve optimizing metadynamics parameters or exploring alternative enhanced sampling techniques to address this issue. Nonetheless, when analyzing the individual simulations, these specific pentanucleotides’ experimental absolute p*K*_a_’s and p*K*_a_ shifts against the trinucleotides were successfully recovered. The binless WHAM procedure assumes overlapping conformational spaces among the different simulations. However, as shown in the results, sampling issues and lack of convergence impacted its predictive ability.

The simulations allowed us to extract some general coupling properties between conformation and protonation in single-strand RNA oligomers. First, the p*K*_a_ of titratable sites is strongly influenced by strand length and, thus, by the content of charged phosphate groups. In longer oligomers with higher phosphate content, an upward shift in the global p*K*_a_ consistent with experiments,^8,10,11^ is observed. Second, stacking interactions of flanking residues can shield phosphate electrostatics and reduce solvent exposure, thereby preventing protonation events in protonable systems and deprotonation events in deprotonable systems. Therefore, stacking interactions and desolvation tend to favor charge neutrality. Meanwhile, at lower pH, protonation events are favored in all systems, leading to increased interactions with neighboring phosphates that simultaneously disrupt stacking interactions and reduce the population of stacked conformations. Third, the *syn/anti* transition of the titratable site also impacts its p*K*_a_ due to its proximity to the phosphate backbone. In shorter strands, close phosphate contacts with the titratable site are dictated by *syn* states, further upshifting the p*K*_a_. Hypothetically, the direction of the shift would be reversed for a titratable site located in a region of the nucleobase that moves farther from the phosphate group upon transitioning to *syn*.

Specifically in deprotonable systems, the method also effectively captures the effect of the flanking residues in strands whose structural dynamics are less sensitive to pH changes, such as rA**G**C/rCA**G**CA. In these cases, each conformational state population is less affected by average charge variations than by stacking-dependent thermodynamic stability. Additionally, our approach can grasp the correlation between multiple simultaneous deprotonation events, as demonstrated for polyU. Simultaneous titration of multiple nucleotides provides a valuable advantage in dissecting the contributions and effects of and upon single sites, a task often difficult for experimental techniques. Our predictions match the experimental p*K*_a_ shift between trimers and pentamers while revealing distinct p*K*_a_ shifts for individual uridines at different topological positions, each exposed to slightly different electrostatic neighbors. These topological effects have been measured experimentally for other sequences^8,12^ corroborating our results that quantitatively highlight that titration correlation is substantial only between nearest neighbors. Overall, our CpHMD simulations robustly reproduced experimental data and provided novel insight into molecular conformations of RNA single-strands that fall outside the scope of experiments.

Regarding the *a posteriori* p*K*^mod^ calibration, two particular points must be noted for the polyU and the guanosine systems. First, the experimental data does not specify a pentamer system representative of polyU. However, the authors discarded the presence of tetra-, tri-, di, and mononucleotides in their polyU experiments. Hence, we assumed that a pentamer system was the minimal model required to predict the experimental p*K*_a_ value. Second, concerning the guanosine systems, a conflict arises from the p*K*_a_ measurements of titratable guanosines: González-Olvera et al.^10^ reported p*K*_a_’s 9.2 and 9.7 for trimer and pentamer, respectively. In contrast, Acharya et al.^12^ reported values of 10.1 and 10.6. For these specific systems, we only considered the NMR measurements of Acharya et al.^12^ for two reasons: they are directly comparable to our RNA systems and the multiple aromatic markers for p*K*_a_ measurements confer higher confidence in the reported values relative to the González-Olvera paper data.

Importantly, our calculations show that the adopted st-CpHMD method does not reproduce the experimental p*K*_a_ shifts from monomers to trimers. However, it correctly reproduces the p*K*_a_ shifts from trimers to pentamers, which is the major goal of the methodology. For this reason, we recalibrated each nucleobase p*K*^mod^ based on their respective trimer simulations, at the expense of having a model that cannot reproduce single nucleosides in water. This correction is similar to that used in amino acid p*K*^mod^ calibration protocols, where pentapeptide constructs are employed, as demonstrated by Machuqueiro.^47^ We consider this a minor defect of the model, and these recalibrated p*K*^mod^ are recommended for use in further simulation studies.

The present work differs from previous studies on nucleic acid CpHMD, which focused on reproducing the p*K*_a_ of nucleosides in water.^27^ Goh et al.^27^ focused on reproducing p*K*_a_ shifts in monophosphorylated nucleotides to validate their methodology, whereas our study incorporates both backbone and flanking interactions, leading to a more comprehensive description of RNA macromolecular environments. Hence, the accuracy of both procedures is not directly comparable. Finally, the enhanced sampling strategy employed here offers improved convergence of the conformational and protonation landscape, which might be more challenging with pH replica exchange methods alone. Although in this work we only tested well-tempered metadynamics, PLUMED integration paves the way for a wide range of enhanced sampling methods based on collective variables.

## Conclusions

Constant-pH molecular dynamics methods are powerful techniques for incorporating titration effects in *in silico* biomolecular studies. In this work, we successfully extended the st-CpHMD methodology to nucleic acids, achieving accurate predictions of protonation states and p*K*_a_ values in short oligonucleotides. The seamless integration of well-tempered metadynamics with PLUMED (CpH-MetaD) enhanced convergence and enabled more efficient sampling of protonation-conformation coupling. Usually, neutral conformations are assumed to be the most thermodynamically stable within the physiological pH range. However, these conformations solely represent part of the free-energy landscape. The strength of CpH methods lies in their ability to capture the protonation-dependent relative probabilities of conformational states, including higher-energy states that might be biologically relevant. These molecular insights are difficult to obtain without explicitly considering protonation effects simultaneously with conformational sampling.

Our results agree with experimental p*K*_a_ shifts across small oligonucleotides, including complex systems like polyU with multiple coupled titrating sites. Despite an initial overestimation of absolute p*K*_a_ values, *a posteriori* p*K*^mod^ corrections improved calibration across all nucleobases in biomolecular environments. These results validate the standard st-CpHMD methodology applied to nucleic acids and the integration of metadynamics in the production MD step. These findings present the CpH-metaD approach as a robust tool for studying pH-dependent processes in nucleic acids, paving the way for investigating larger, biologically relevant RNA systems, such as ribozymes or RNA-protein complexes, where pH fluctuations play an important role in function and stability. This implementation is being tested in more structured systems, such as pH-dependent duplexes. In the future, this methodology is best suited to biological systems with pH-dependent conformational transitions, such as the 5′ terminal stem-loop of SARS-COV-2, whose two-state transitions depend upon the protonation of an adenine nucleotide near a bulge to form a noncanonical A^+^-C wobble base pair. Future work may focus on improving the parameters of metadynamics and Poisson–Boltzmann calculations or applying other enhanced sampling techniques to improve p*K*_a_ accuracy and the sampling of conformational or protonation space in more complex systems.

## Data Availability

The GROMACS package is freely available software to perform MD simulations and can be downloaded at https://manual.gromacs.org/documentation/2020.1/download.html. PyMOL v2.5 is also free software for molecular visualization and generating high-quality images. It can be downloaded from https://pymol.org/2. The WHAM module is available at https://bussilab.github.io/doc-py-bussilab/bussilab/wham.html. The modified nucleic acid parameters for the st-CpHMD are available at https://github.com/Tomfersil/CpH-MetaD. All the input scripts are available at https://github.com/Tomfersil/CpH-MetaD. PLUMED input files are also available on PLUMED-NEST^67^ with ID 24.026. As part of the PLUMED Tutorials consortium^91^ a tutorial on setting up and analyzing constant-pH metadynamics simulations is available on the https://www.plumed-tutorials.org/ Web site under ID 24.020.

## Supporting Information Available

Charge set derivations for all nucleobases using a RESP procedure (Tables S1–S4); simulation settings for all oligomer systems (Table S5); ^3^J scalar coupling values for all nucleosides from modular, χOL3, and experimental NMR data (Tables S6–S9); χ angle syn and anti populations of each nucleotide using χOL3 and modular force field (Table S10); p*K*_a_ values for the titratable sites of all simulated oligomer systems (Table S11); reference experimental p*K*_a_ values and final p*K*^mod^ values for the titratable nucleobases (Table S12); individual pKa values for the titratable r**UUUUU** sites (Table S13); time series of the χ angle between the adenine nucleobase and the sugar using CpHMD and CpH-metaD simulations (Figure S1); 2D free energy maps and titration curves of the titratable cytidine and guanosine single-strand oligomers (Figures S2 and S3); cartoon representations of representative conformations of each studied oligomer sequence (Figures S4–S10); 2D free energy maps at different pH range values for each studied oligomer sequence (Figures S11–S22); base-stacking dependent titration curves and p*K*_a_ values of all studied systems except r**UUUUU** (Figure S23); pairwise protonation cooperativity matrix of r**UUUUU** at the pH 9.5 and 10 (Figure S24) (PDF)

## References

[ref1] Putnam R. W. Cell Physiology Source Book, 4 th ed., Sperelakis N.; Academic Press: San Diego, 2012; pp. 303–321

[ref2] Aoi W.; Marunaka Y. Importance of pH homeostasis in metabolic health and diseases: crucial role of membrane proton transport. Biomed Res. Int. 2014, 2014, 598986 10.1155/2014/598986. 25302301 PMC4180894

[ref3] Chen F.; Zhuang X.; Lin L.; Yu P.; Wang Y.; Shi Y.; Hu G.; Sun Y. New horizons in tumor microenvironment biology: challenges and opportunities. BMC Med. 2015, 13 (1), 45 10.1186/s12916-015-0278-7. 25857315 PMC4350882

[ref4] Wang M.; Zhao J.; Zhang L.; Wei F.; Lian Y.; Wu Y.; Gong Z.; Zhang S.; Zhou J.; Cao K.; et al. Role of tumor microenvironment in tumorigenesis. J. Cancer 2017, 8 (5), 761–773. 10.7150/jca.17648. 28382138 PMC5381164

[ref5] Legault P.; Pardi A. Unusual dynamics and p K a shift at the active site of a lead-dependent ribozyme. J. Am. Chem. Soc. 1997, 119, 6621–6628. 10.1021/ja9640051.

[ref6] Jones E. L.; Mlotkowski A. J.; Hebert S. P.; Schlegel H. B.; Chow C. S. Calculations of p K a values for a series of naturally occurring modified nucleobases. J. Phys. Chem. A 2022, 126, 1518–1529. 10.1021/acs.jpca.1c10905. 35201779

[ref7] Izatt R. M.; Christensen J. J.; Rytting J. H. Sites and thermodynamic quantities associated with proton and metal ion interaction with ribonucleic acid, deoxyribonucleic acid, and their constituent bases, nucleosides, and and nucleotides. Chem. Rev. 1971, 71, 439–481. 10.1021/cr60273a002. 5126179

[ref8] Acharya P.; Cheruku P.; Chatterjee S.; Acharya S.; Chattopadhyaya J. Measurement of Nucleobase p*K*_a_ Values in Model Mononucleotides Shows RNA- RNA Duplexes To Be More Stable than DNA- DNA Duplexes. J. Am. Chem. Soc. 2004, 126, 2862–2869. 10.1021/ja0386546. 14995203

[ref9] Clauwaert J.; Stockx J. Interactions of polynucleotides and their components: I. Dissociation constants of the bases and their derivatives. Z. Naturforsch. B 1968, 23, 25–30. 10.1515/znb-1968-0105. 4387850

[ref10] González-Olvera J. C.; Martínez-Reyes J.; Gonzalez-Jasso E.; Pless R. C. Determination of pKa values for deprotonable nucleobases in short model oligonucleotides. Biophys. Chem. 2015, 206, 58–65. 10.1016/j.bpc.2015.07.001. 26188860

[ref11] González-Olvera J. C.; Durec M.; Marek R.; Fiala R.; Morales-García M. D. R. J.; González-Jasso E.; Pless R. C. Protonation of Nucleobases in Single-and Double-Stranded DNA. ChemBiochem 2018, 19, 2088–2098. 10.1002/cbic.201800310. 30073767

[ref12] Acharya S.; Barman J.; Cheruku P.; Chatterjee S.; Acharya P.; Isaksson J.; Chattopadhyaya J. Significant pKa Perturbation of Nucleobases Is an Intrinsic Property of the Sequence Context in DNA and RNA. J. Am. Chem. Soc. 2004, 126, 8674–8681. 10.1021/ja048484c. 15250719

[ref13] Acharya P.; Chattopadhyaya J. Electrostatic cross-modulation of the pseudoaromatic character in single-stranded RNA by nearest-neighbor interactions. Pure Appl. Chem. 2005, 77, 291–311. 10.1351/pac200577010291.

[ref14] Collin D.; Gehring K. Stability of chimeric DNA/RNA cytosine tetrads: implications for i-motif formation by RNA. J. Am. Chem. Soc. 1998, 120, 4069–4072. 10.1021/ja973346r.

[ref15] Guéron M.; Leroy J.-L. The i-motif in nucleic acids. Curr. Opin. Struct. Biol. 2000, 10, 326–331. 10.1016/S0959-440X(00)00091-9. 10851195

[ref16] Rhee S.; Han Z.-J.; Liu K.; Miles H. T.; Davies D. R. Structure of a triple helical DNA with a triplex- duplex junction. Biochemistry 1999, 38, 16810–16815. 10.1021/bi991811m. 10606513

[ref17] Hu Y.; Cecconello A.; Idili A.; Ricci F.; Willner I. Triplex DNA nanostructures: from basic properties to applications. Angew. Chem., Int. Ed.Angew. Chem., Int. Ed. 2017, 56, 15210–15233. 10.1002/anie.201701868. 28444822

[ref18] Mariottini D.; Del Giudice D.; Ercolani G.; Di Stefano S.; Ricci F. Dissipative operation of pH-responsive DNA-based nanodevices. Chem. Sci. 2021, 12, 11735–11739. 10.1039/D1SC03435A. 34659709 PMC8442697

[ref19] Farag N.; Mattossovich R.; Merlo R.; Nierzwicki Ł.; Palermo G.; Porchetta A.; Perugino G.; Ricci F. Folding-upon-Repair DNA Nanoswitches for Monitoring the Activity of DNA Repair Enzymes. Angew. Chem., Int. Ed.Angew. Chem., Int. Ed. 2021, 133, 7359–7365. 10.1002/ange.202016223. PMC8783695 33415794

[ref20] Cecconello A.; Magro M.; Vianello F.; Simmel F. C. Rational design of hybrid DNA–RNA triplex structures as modulators of transcriptional activity in vitro. Nucleic Acids Res. 2022, 50, 13172–13182. 10.1093/nar/gkac1131. 36537227 PMC9825147

[ref21] Miao D.; Yu Y.; Chen Y.; Liu Y.; Su G. Facile construction of i-Motif DNA-conjugated gold nanostars as near-infrared and pH dual-responsive targeted drug delivery systems for combined cancer therapy. Mol. Pharm. 2020, 17, 1127–1138. 10.1021/acs.molpharmaceut.9b01159. 32092274

[ref22] Idili A.; Vallée-Bélisle A.; Ricci F. Programmable pH-triggered DNA nanoswitches. J. Am. Chem. Soc. 2014, 136, 5836–5839. 10.1021/ja500619w. 24716858

[ref23] Mariottini D.; Idili A.; Nijenhuis M. A.; Ercolani G.; Ricci F. Entropy-based rational modulation of the pKa of a synthetic pH-dependent nanoswitch. J. Am. Chem. Soc. 2019, 141, 11367–11371. 10.1021/jacs.9b04168. 31296004

[ref24] Sponer J.; Bussi G.; Krepl M.; Banáš P.; Bottaro S.; Cunha R. A.; Gil-Ley A.; Pinamonti G.; Poblete S.; Jurecka P.; et al. RNA structural dynamics as captured by molecular simulations: a comprehensive overview. Chem. Rev. 2018, 118, 4177–4338. 10.1021/acs.chemrev.7b00427. 29297679 PMC5920944

[ref25] Kong X.; Brooks C. L. III *λ*-dynamics: A new approach to free energy calculations. J. Chem. Phys. 1996, 105, 2414–2423. 10.1063/1.472109.

[ref26] Lee M. S.; Salsbury F. R.; Brooks C. L. III Constant-pH molecular dynamics using continuous titration coordinates. Proteins: struct., Funct., Bioinf.Proteins: struct., Funct., Bioinf. 2004, 56, 738–752. 10.1002/prot.20128. 15281127

[ref27] Goh G. B.; Knight J. L.; Brooks C. L. Constant pH molecular dynamics simulations of nucleic acids in explicit solvent. J. Chem. Theory Comput. 2012, 8, 36–46. 10.1021/ct2006314. 22337595 PMC3277849

[ref28] Goh G. B.; Hulbert B. S.; Zhou H.; Brooks C. L. III Constant pH molecular dynamics of proteins in explicit solvent with proton tautomerism. Proteins: struct., Funct., Bioinf.Proteins: struct., Funct., Bioinf. 2014, 82, 1319–1331. 10.1002/prot.24499. PMC4394622 24375620

[ref29] Huang Y.; Chen W.; Wallace J. A.; Shen J. All-atom continuous constant pH molecular dynamics with particle mesh Ewald and titratable water. J. Chem. Theory Comput. 2016, 12, 5411–5421. 10.1021/acs.jctc.6b00552. 27709966 PMC5713900

[ref30] Aho N.; Buslaev P.; Jansen A.; Bauer P.; Groenhof G.; Hess B. Scalable Constant pH Molecular Dynamics in GROMACS. J. Chem. Theory Comput. 2022, 18 (10), 6148–6160. 10.1021/acs.jctc.2c00516. 36128977 PMC9558312

[ref31] Donnini S.; Tegeler F.; Groenhof G.; Grubmüller H. Constant pH molecular dynamics in explicit solvent with *λ*-dynamics. J. Chem. Theory Comput. 2011, 7, 1962–1978. 10.1021/ct200061r. 21687785 PMC3114466

[ref32] Wallace J. A.; Shen J. K. Charge-leveling and proper treatment of long-range electrostatics in all-atom molecular dynamics at constant pH. J. Chem. Phys. 2012, 137 (18), 184105 10.1063/1.4766352. 23163362 PMC3511335

[ref33] Goh G. B.; Knight J. L.; Brooks C. L. III Toward accurate prediction of the protonation equilibrium of nucleic acids. J. Phys. Chem. Lett. 2013, 4, 760–766. 10.1021/jz400078d. 23526474 PMC3601767

[ref34] Khandogin J.; Brooks C. L. III Constant pH molecular dynamics with proton tautomerism. Biophys. J. 2005, 89, 141–157. 10.1529/biophysj.105.061341. 15863480 PMC1366513

[ref35] Tang C. L.; Alexov E.; Pyle A. M.; Honig B. Calculation of pKas in RNA: on the structural origins and functional roles of protonated nucleotides. J. Mol. Biol. 2007, 366, 1475–1496. 10.1016/j.jmb.2006.12.001. 17223134

[ref36] Barroso da Silva F. L.; Derreumaux P.; Pasquali S. Fast coarse-grained model for RNA titration. J. Chem. Phys. 2017, 146 (3), 035101 10.1063/1.4972986. 28109220

[ref37] Pasquali S.; Frezza E.; Barroso da Silva F. Coarse-grained dynamic RNA titration simulations. Interface Focus 2019, 9, 20180066 10.1098/rsfs.2018.0066. 31065339 PMC6501344

[ref38] Knight J. L.; Brooks C. L. III Multisite *λ* dynamics for simulated structure–activity relationship studies. J. Chem. Theory Comput. 2011, 7, 2728–2739. 10.1021/ct200444f. 22125476 PMC3223982

[ref39] Itoh S. G.; Damjanović A.; Brooks B. R. pH replica-exchange method based on discrete protonation states. Proteins: struct., Funct., Bioinf.Proteins: struct., Funct., Bioinf. 2011, 79, 3420–3436. 10.1002/prot.23176. PMC3373023 22002801

[ref40] Baptista A. M.; Teixeira V. H.; Soares C. M. Constant-pH molecular dynamics using stochastic titration. J. Chem. Phys. 2002, 117, 4184–4200. 10.1063/1.1497164.

[ref41] Burgi R.; Kollman P. A.; van Gunsteren W. F. Simulating proteins at constant pH: An approach combining molecular dynamics and Monte Carlo simulation. Proteins: struct., Funct., Bioinf.Proteins: struct., Funct., Bioinf. 2002, 47, 469–480. 10.1002/prot.10046. 12001225

[ref42] Dlugosz M.; Antosiewicz J. M. Constant-pH molecular dynamics simulations: a test case of succinic acid. Chem. Phys. 2004, 302, 161–170. 10.1016/j.chemphys.2004.03.031.

[ref43] Machuqueiro M.; Baptista A. M. Constant-pH Molecular Dynamics with Ionic Strength Effects: Protonation–Conformation Coupling in Decalysine. J. Phys. Chem. B 2006, 110, 2927–2933. 10.1021/jp056456q. 16471903

[ref44] Machuqueiro M.; Baptista A. M. Acidic range titration of HEWL using a constant-pH molecular dynamics method. Proteins: struct., Funct., Bioinf.Proteins: struct., Funct., Bioinf. 2008, 72, 289–298. 10.1002/prot.21923. 18214978

[ref45] Teixeira V. H.; Vila-Viçosa D.; Reis P. B. P. S.; Machuqueiro M. p*K*_a_ Values of Titrable Amino Acids at the Water/Membrane Interface. J. Chem. Theory Comput. 2016, 12, 930–934. 10.1021/acs.jctc.5b01114. 26863409

[ref46] Sequeira J. G.; Rodrigues F. E.; Silva T. G.; Reis P. B.; Machuqueiro M. Extending the stochastic titration CpHMD to CHARMM36m. J. Phys. Chem. B 2022, 126, 7870–7882. 10.1021/acs.jpcb.2c04529. 36190807 PMC9776569

[ref47] Machuqueiro M.; Baptista A. M. Is the prediction of p*K*_a_ values by constant-pH molecular dynamics being hindered by inherited problems?. Proteins: Struct., Funct., Bioinf.Proteins: Struct., Funct., Bioinf. 2011, 79, 3437–3447. 10.1002/prot.23115. 22072522

[ref48] Teixeira V. H.; Cunha C. C.; Machuqueiro M.; Oliveira A. S. F.; Victor B. L.; Soares C. M.; Baptista A. M. On the Use of Different Dielectric Constants for Computing Individual and Pairwise Terms in Poisson-Boltzmann Studies of Protein Ionization Equilibrium. J. Phys. Chem. B 2005, 109, 14691–14706. 10.1021/jp052259f. 16852854

[ref49] Kührová P.; Banas P.; Best R. B.; Sponer J.; Otyepka M. Computer folding of RNA tetraloops? Are we there yet?. J. Chem. Theory Comput. 2013, 9, 2115–2125. 10.1021/ct301086z. 26583558

[ref50] Haldar S.; Kührová P.; Banas P.; Spiwok V.; Sponer J.; Hobza P.; Otyepka M. Insights into stability and folding of GNRA and UNCG tetraloops revealed by microsecond molecular dynamics and well-tempered metadynamics. J. Chem. Theory Comput. 2015, 11, 3866–3877. 10.1021/acs.jctc.5b00010. 26574468

[ref51] Mlynsky V.; Janeček M.; Kuhrova P.; Frohlking T.; Otyepka M.; Bussi G.; Banas P.; Sponer J. Toward convergence in folding simulations of RNA tetraloops: Comparison of enhanced sampling techniques and effects of force field modifications. J. Chem. Theory Comput. 2022, 18, 2642–2656. 10.1021/acs.jctc.1c01222. 35363478

[ref52] Bussi G.; Laio A. Using metadynamics to explore complex free-energy landscapes. Nat. Rev. Phys. 2020, 2, 200–212. 10.1038/s42254-020-0153-0.

[ref53] Cornell W. D.; Cieplak P.; Bayly C. I.; Gould I. R.; Merz K. M.; Ferguson D. M.; Spellmeyer D. C.; Fox T.; Caldwell J. W.; Kollman P. A. A second generation force field for the simulation of proteins, nucleic acids, and organic molecules. J. Am. Chem. Soc. 1995, 117, 5179–5197. 10.1021/ja00124a002.

[ref54] Pérez A.; Marchán I.; Svozil D.; Sponer J.; Cheatham T. E.; Laughton C. A.; Orozco M. Refinement of the AMBER force field for nucleic acids: improving the description of *α*/*γ* conformers. Biophys. J. 2007, 92, 3817–3829. 10.1529/biophysj.106.097782. 17351000 PMC1868997

[ref55] Zgarbová M.; Otyepka M.; Šponer J.; Mládek A.; Banáš P.; Cheatham T. E. III; Jurecka P. Refinement of the Cornell et al. nucleic acids force field based on reference quantum chemical calculations of glycosidic torsion profiles. J. Chem. Theory Comput. 2011, 7, 2886–2902. 10.1021/ct200162x. 21921995 PMC3171997

[ref56] Izadi S.; Anandakrishnan R.; Onufriev A. V. Building water models: a different approach. J. Phys. Chem. Lett. 2014, 5, 3863–3871. 10.1021/jz501780a. 25400877 PMC4226301

[ref57] Bayly C. I.; Cieplak P.; Cornell W. D.; Kollman P. A. A Well Behaved Electrostatic Based Method Using Charge Restraints For Deriving Atomic Charges: The RESP Model. J. Phys. Chem. 1993, 97, 10269–10280. 10.1021/j100142a004.

[ref58] Aduri R.; Psciuk B. T.; Saro P.; Taniga H.; Schlegel H. B.; SantaLucia J. AMBER force field parameters for the naturally occurring modified nucleosides in RNA. J. Chem. Theory Comput. 2007, 3, 1464–1475. 10.1021/ct600329w. 26633217

[ref59] Frisch M. J.; Trucks G. W.; Schlegel H. B.; Scuseria G. E.; Robb M. A.; Cheeseman J. R.; Scalmani G.; Barone V.; Petersson G. A.; Nakatsuji H., Gaussian 16 Revision C.01.; Gaussian Inc.: Wallingford CT, 2016.

[ref60] Colominas C.; Luque F. J.; Orozco M. Tautomerism and protonation of guanine and cytosine. Implications in the formation of hydrogen-bonded complexes. J. Am. Chem. Soc. 1996, 118, 6811–6821. 10.1021/ja954293l.

[ref61] Lorentz H. A. Ueber die Anwendung des Satzes vom Virial in der kinetischen Theorie der Gase. Ann. Phys. 1881, 248, 127–136. 10.1002/andp.18812480110.

[ref62] Berthelot D. Sur le mélange des gaz. Compt. Rendus 1898, 126, 15.

[ref63] Schrödinger, LLC PyMOL The PyMOL Molecular Graphics System, Version 3.1; Schrödinger, LLC.

[ref64] Huang M.; Giese T. J.; Lee T.-S.; York D. M. Improvement of DNA and RNA sugar pucker profiles from semiempirical quantum methods. J. Chem. Theory Comput. 2014, 10, 1538–1545. 10.1021/ct401013s. 24803866 PMC3985690

[ref65] Abraham M. J.; Murtola T.; Schulz R.; Páll S.; Smith J. C.; Hess B.; Lindahl E. GROMACS: High performance molecular simulations through multi-level parallelism from laptops to supercomputers. SoftwareX 2015, 1–2, 19–25. 10.1016/j.softx.2015.06.001.

[ref66] Bauer P.; Hess B.; Lindahl E. GROMACS 2022 Manual, 2022; Zendo.

[ref67] Promoting transparency and reproducibility in enhanced molecular simulations. Nat. Methods 2019, 16 (8), 670–673. 10.1038/s41592-019-0506-8. 31363226

[ref68] Tribello G. A.; Bonomi M.; Branduardi D.; Camilloni C.; Bussi G. PLUMED 2: New feathers for an old bird. Comput. Phys. Commun. 2014, 185, 604–613. 10.1016/j.cpc.2013.09.018.

[ref69] Bussi G.; Donadio D.; Parrinello M. Canonical sampling through velocity rescaling. J. Chem. Phys. 2007, 126 (1), 014101 10.1063/1.2408420. 17212484

[ref70] Bernetti M.; Bussi G. Pressure control using stochastic cell rescaling. J. Chem. Phys. 2020, 153 (11), 114107 10.1063/5.0020514. 32962386

[ref71] Rocchia W.; Sridharan S.; Nicholls A.; Alexov E.; Chiabrera A.; Honig B. Rapid grid-based construction of the molecular surface and the use of induced surface charge to calculate reaction field energies: Applications to the molecular systems and geometric objects. J. Comput. Chem. 2002, 23, 128–137. 10.1002/jcc.1161. 11913378

[ref72] Teixeira V. H.; Vila-Viçosa D.; Baptista A. M.; Machuqueiro M. Protonation of DMPC in a Bilayer Environment Using a Linear Response Approximation. J. Chem. Theory Comput. 2014, 10, 2176–2184. 10.1021/ct5000082. 26580542

[ref73] Vila-Viçosa D.; Teixeira V. H.; Baptista A. M.; Machuqueiro M. Constant-pH MD simulations of an oleic acid bilayer. J. Chem. Theory Comput. 2015, 11, 2367–2376. 10.1021/acs.jctc.5b00095. 26574431

[ref74] Santos H. A.; Vila-Viçosa D.; Teixeira V. H.; Baptista A. M.; Machuqueiro M. Constant-pH MD simulations of DMPA/DMPC lipid bilayers. J. Chem. Theory Comput. 2015, 11, 5973–5979. 10.1021/acs.jctc.5b00956. 26588046

[ref75] Baptista A. M.; Soares C. M. Some Theoretical and Computational Aspects of the Inclusion of Proton Isomerism in the Protonation Equilibrium of Proteins. J. Phys. Chem. B 2001, 105, 293–309. 10.1021/jp002763e.

[ref76] Barducci A.; Bussi G.; Parrinello M. Well-tempered metadynamics: a smoothly converging and tunable free-energy method. Phys. Rev. Lett. 2008, 100, 020603 10.1103/PhysRevLett.100.020603. 18232845

[ref77] Bottaro S.; Di Palma F.; Bussi G. The role of nucleobase interactions in RNA structure and dynamics. Nucleic Acids Res. 2014, 42, 13306–13314. 10.1093/nar/gku972. 25355509 PMC4245972

[ref78] Bottaro S.; Banáš P.; Sponer J.; Bussi G. Free energy landscape of GAGA and UUCG RNA tetraloops. J. Phys. Chem. Lett. 2016, 7, 4032–4038. 10.1021/acs.jpclett.6b01905. 27661094

[ref79] Ancian B. Annual Reports on NMR Spectroscopy; Elsevier, 2010, Vol. 69, pp. 39–143.

[ref80] Munzarová M. L.; Sklenář V. DFT analysis of NMR scalar interactions across the glycosidic bond in DNA. J. Am. Chem. Soc. 2003, 125, 3649–3658. 10.1021/ja028931t. 12643728

[ref81] Condon D. E.; Kennedy S. D.; Mort B. C.; Kierzek R.; Yildirim I.; Turner D. H. Stacking in RNA: NMR of four tetramers benchmark molecular dynamics. J. Chem. Theory Comput. 2015, 11, 2729–2742. 10.1021/ct501025q. 26082675 PMC4463549

[ref82] Branduardi D.; Bussi G.; Parrinello M. Metadynamics with adaptive Gaussians. J. Chem. Theory Comput. 2012, 8, 2247–2254. 10.1021/ct3002464. 26588957

[ref83] Souaille M.; Roux B. Extension to the weighted histogram analysis method: combining umbrella sampling with free energy calculations. Comput. Phys. Commun. 2001, 135, 40–57. 10.1016/S0010-4655(00)00215-0.

[ref84] Shirts M. R.; Chodera J. D. Statistically optimal analysis of samples from multiple equilibrium states. J. Chem. Phys. 2008, 129 (12), 124105 10.1063/1.2978177. 19045004 PMC2671659

[ref85] Tan Z.; Gallicchio E.; Lapelosa M.; Levy R. M. Theory of binless multi-state free energy estimation with applications to protein-ligand binding. J. Chem. Phys. 2012, 136 (14), 144102 10.1063/1.3701175. 22502496 PMC3339880

[ref86] Benjamini Y.; Hochberg Y. Controlling the false discovery rate: a practical and powerful approach to multiple testing. J. R. Stat. Soc. 1995, 57, 289–300. 10.1111/j.2517-6161.1995.tb02031.x.

[ref87] Tanford C. Physical chemistry of macromolecules; John Wiley & Sons, INC., 1961; pp. 572–573.

[ref88] Di Russo N. V.; Martí M. A.; Roitberg A. E. Underlying thermodynamics of pH-dependent allostery. J. Phys. Chem. B 2014, 118, 12818–12826. 10.1021/jp507971v. 25318010

[ref89] Swails J. M.; York D. M.; Roitberg A. E. Constant pH replica exchange molecular dynamics in explicit solvent using discrete protonation states: implementation, testing, and validation. J. Chem. Theory Comput. 2014, 10, 1341–1352. 10.1021/ct401042b. 24803862 PMC3985686

[ref90] Vila-Viçosa D.; Silva T. F.; Slaybaugh G.; Reshetnyak Y. K.; Andreev O. A.; Machuqueiro M. Membrane-Induced p*K*_a_ Shifts in wt-pHLIP and Its L16H Variant. J. Chem. Theory Comput. 2018, 14, 3289–3297. 10.1021/acs.jctc.8b00102. 29733633 PMC6287259

[ref91] Tribello G. A.; Bonomi M.; Bussi G.; Camilloni C.; Armstrong B. I.; Arsiccio A.; Aureli S.; Ballabio F.; Bernetti M.; Bonati L.; et al. PLUMED Tutorials: A collaborative, community-driven learning ecosystem. J. Chem. Phys. 2025, 162 (9), 092501 10.1063/5.0251501. 40035582 PMC12317779

