# Supplementary material for: Characterizing RNA Oligomers Using Stochastic Titration Constant-pH Metadynamics Simulations
Source: J Chem Inf Model. 2025 Mar 18;65(7):3568–80. doi: 10.1021/acs.jcim.4c02185 (PMC12004511; doi:10.1021/acs.jcim.4c02185)
Supplement: Supplementary file 1 — ci4c02185_si_001.pdf [file ci4c02185_si_001.pdf]

Characterizing RNA oligomers using Stochastic Titration Constant-pH Metadynamics simulations

Tomás F. D. Silva* and Giovanni Bussi

Scuola Internazionale Superiore di Studi Avanzati, 34136 Trieste, Italy

E-mail: tfernand@sissa.it

Supporting Information

March 4, 2025

Table S1: Charge set derivation for the neutral and charged states of the adenosine nucleobase. The protonation event occurs at the N1 atom. The charges on C1'(*) and H1'(*) are preserved in both states within the sugar block of the modified force field. The RESP charges on the N9 and the equivalenced methyl moiety were manually curated to offset the modularity modification of the sugar moiety. Charge rebalancing of N9(**) in both states is necessary for the modularity correction, ensuring that a full integer charge is restricted solely to the nucleobase. The equal charge on both states results from variations observed in the RESP procedure. The remaining charges of the deprotonated state are equal to those in the χOL3 force field except for the N9(**).

**Atom**	Neutral Adenosine	Charged (+) Adenosine
C1'	-0.0840*	-0.0840
H1'	0.2000*	0.2000
N9	0.1529**	0.1529
C8	0.2006	0.2606
H8	0.1553	0.1853
N7	-0.6073	-0.6373
C5	0.0515	0.1115
C6	0.7009	0.6209
N6	-0.9019	-0.8419
H61	0.4115	0.4515
H62	0.4115	0.4515
N1	-0.7615	-0.6515
H11	0.0000	0.4900
C2	0.5875	0.5375
H2	0.0473	0.1273
N3	-0.6997	-0.6397
C4	0.3053	0.4753

Table S2: Charge set derivation for the neutral and charged states of the cytidine nucleobase. The protonation event occurs at the N3 atom. The charges on C1'(*) and H1'(*) are preserved in both states within the sugar block of the modified force field. The RESP charges on the N1 and the equivalenced methyl moiety were manually curated to offset the modularity modification of the sugar moiety. Charge rebalancing of N1(**) in both states is necessary for the modularity correction, ensuring that a full integer charge is restricted solely to the nucleobase. The remaining charges of the deprotonated state are equal to those in the χOL3 force field except for the N1(**).

**Atom**	Neutral Cytidine	Charged (+) Cytidine
C1'	-0.0840*	-0.0840
H1'	0.2000*	0.2000
N1	0.045**	0.1726
C6	0.2006	-0.2147
H6	0.1553	0.2858
C5	-0.6073	-0.0815
H5	0.0515	0.1528
C4	0.7009	0.3260
N4	-0.9019	-0.7730
H41	0.4115	0.4434
H42	0.4115	0.4434
N3	-0.7584	-0.0484
H3	0.0000	0.2900
C2	0.7538	0.4013
O2	-0.6252	-0.3977

Table S3: Charge set derivation for the neutral and charged states of the uridine nucleobase. The protonation event occurs an the N3 atom. The charges on C1'(*) and H1'(*) are preserved in both states within the sugar block of the modified force field. The RESP charges on the N1 and the equivalenced methyl moiety were manually curated to offset the modularity modification of the sugar moiety. Charge rebalancing of N1(**) on both states is necessary for the modularity correction, ensuring that a full integer charge is restricted solely to the nucleobase. The remaining charges of the deprotonated state are equal to those in the χOL3 force field except for the N1(**).

**Atom**	Neutral Uridine	Charged (-) Uridine
C1'	-0.0840*	-0.0840
H1'	0.2000*	0.2000
N1	0.1756**	0.0361
C6	-0.1126	-0.1104
H6	0.2188	0.1430
C5	-0.3635	-0.4931
H5	0.1811	0.1492
C4	0.5952	0.7924
O4	-0.5761	-0.7397
N3	-0.3549	-0.7155
H3	0.3154	0.000
C2	0.4687	0.6290
O2	-0.5477	-0.6910

Table S4: Charge set derivation for the neutral and charged states of the guanosine nucleobase. The protonation event occurs at the N1 atom. The charges on C1'(*) and H1'(*) are preserved in both states within the sugar block of the modified force field. The RESP charges on the N9 and the equivalenced methyl moiety were manually curated to offset the modularity modification of the sugar moiety. Charge rebalancing of N9(**) on both states is necessary for the modularity correction, ensuring that a full integer charge is restricted solely to the nucleobase. The remaining charges of the deprotonated state are equal to those in the χOL3 force field except for the N9(**).

**Atom**	Neutral Guanosine	Charged (-) Guanosine
C1'	-0.0840*	-0.0840
H1'	0.2000*	0.2000
N9	0.15296*	0.1445
C8	0.1374	0.0692
H8	0.164	0.0995
N7	-0.5709	-0.5995
C5	0.1744	0.1032
C6	0.4770	0.5734
O6	-0.5597	-0.6882
N1	-0.4787	-0.6634
H1	0.3424	0.000
C2	0.7657	0.7628
N2	-0.9672	-0.9667
H21	0.4364	0.3908
H22	0.4364	0.3908
N3	-0.6323	-0.7245
C4	0.1222	0.1081

Table S5: Table of simulation settings for all oligomer systems. All simulations were performed at 300 K using the CpH-metaD approach except for the r**UUUUU** system (* performed with st-CpHMD). The collective variables (CV) used were: the χ angle between the titratable nucleobase (in bold) and its sugar moiety, and the eRMSD of the strand relative to a fully stacked system configuration.

Oligomers	Sim. time (μs)	pH range	Ionic Strength (M)	Bias factor
rU**A**U	1.5	[3.0,3.5,4.0]	0.01	8
rUU**A**UU	3	[3.5,4.0,4.5]	0.01	15
rU**C**U	2	[4.0,4.5,5.0]	0.01	8
rUU**C**UU	3	[4.0,4.5,5.0,5.5]	0.01	15
rA**G**C	1.5	[9.0,9.5,10.0,11.0]	0.1	8
rCA**G**CA	3	[9.0,9.5,10.0,11.0]	0.1	15
rC**U**C	1.5	[9.0,9.5,10.0]	0.1	8
r**UUUUU***	1.5	[9.5,10.0,10.5]	0.1	-

Table S6: ^{3}J scalar coupling values for the neutral adenosine nucleoside were obtained using the modular charge set, the original χOL3, and NMR experimental data.[79] The total RMSE is presented for both the modular charge set and the standard χOL3 force field. All standard errors obtained were smaller than 0.05 Hz.

Adenosine	$J_{HC} (Hz)$		J_{HH} (Hz)					
Source	χ	χ'	$\nu 1$	$\nu 2$	$\nu 3$	γ'	γ''	RMSE (Hz)
Modular	4.33	3.50	7.63	5.20	4.46	3.43	1.67	1.05
χOL3	4.27	3.51	7.88	5.11	4.21	3.41	1.65	1.07
NMR	3.6	3.9	6.0	5.0	3.4	3.0	3.4	-

Table S7: ^{3}J scalar coupling values for the neutral guanosine nucleoside were obtained using the modular charge set, the original χOL3, and NMR experimental data.[79] The total RMSE is presented for both the modular charge set and the standard χOL3 force field. All standard errors obtained were smaller than 0.05 Hz.

Guanosine	$J_{HC} (Hz)$		J_{HH} (Hz)					
Source	χ	χ'	$\nu 1$	$\nu 2$	$\nu 3$	γ'	γ''	RMSE (Hz)
Modular	3.92	3.54	6.30	5.24	5.90	3.33	1.59	1.23
χOL3	3.86	3.55	6.53	5.18	5.71	3.31	1.55	1.20
NMR	2.5	4.5	5.5	5.1	3.9	3.2	3.3	-

Table S8: ^{3}J scalar coupling values for the neutral cytidine nucleoside were obtained using the modular charge set, the original χOL3, and NMR experimental data.[79] The total RMSE is presented for both the modular charge set and the standard χOL3 force field. All standard errors obtained were smaller than 0.05 Hz.

Cytidine	$J_{HC} (Hz)$		J_{HH} (Hz)					
Source	χ	χ'	$\nu 1$	$\nu 2$	$\nu 3$	γ'	γ''	RMSE (Hz)
Modular	2.49	3.68	6.69	5.22	6.19	3.55	1.98	1.50
χOL3	2.35	3.62	6.87	5.16	5.98	3.55	1.93	1.55
NMR	1.9	3.3	3.6	5.0	5.8	2.8	4.2	-

Table S9: ^{3}J scalar coupling values for the neutral uridine nucleoside were obtained using the modular charge set, the original χOL3, and NMR experimental data.[79] The total RMSE is presented for the modular charge set and the standard χOL3 force field. All standard errors obtained were smaller than 0.05 Hz.

Uridine	$J_{HC} (Hz)$		J_{HH} (Hz)					
Source	χ	χ'	$\nu 1$	$\nu 2$	$\nu 3$	γ'	γ''	RMSE (Hz)
Modular	2.78	3.67	6.27	5.29	6.73	3.62	2.15	1.22
χOL3	2.72	3.65	6.71	5.17	6.21	3.51	2.01	1.30
NMR	2.3	3.6	4.3	5.3	5.6	3.0	4.3	-

Table S10: Table of syn ($0 \leq \chi \leq +\frac{\pi}{2}$) and anti populations for each nucleotide. 100 ns wt-metadynamics MD simulations were initially performed using the χOL3 force field. The chosen collective variables were the sugar puckering and the χ angle. Then, a new topology was generated for the modified modular force field, and a rerun procedure was employed to recompute the populations with the new charge set.

Nucleotides	Syn		Anti	
	OL3	Modular	OL3	Modular
Adenosine	0.74	0.76	0.26	0.24
Cytidine	0.20	0.23	0.8	0.77
Guanosine	0.65	0.65	0.35	0.35
Uridine	0.14	0.13	0.86	0.87

Table S11: Table of pK_a values for the titratable sites (in bold) of all simulated oligomer systems obtained before the final calibration step. The columns refer to the reference experimental pK_a values under different experimental conditions, the pK_a values estimated from the individual average protonations of all CpH simulations fitted to the Henderson-Hasselbalch (HH) equation, and the pK_a values estimated using the WHAM procedure described in the Methods section. The average protonations used for the HH fit pK_a estimation were obtained via block analysis by splitting the equilibrated trajectory into four equally sized blocks.

Oligomers	Reference pK_a	HH fit pK_a	WHAM fit pK_a
rU**A**U	3.48[11]	3.86 ± 0.16	3.97 ± 0.17
rUU**A**UU	3.80[11]	4.31 ± 0.28	4.32 ± 0.13
rU**C**U	4.24[11]	4.64 ± 0.11	4.57 ± 0.13
rUU**C**UU	4.90[11]	5.07 ± 0.12	4.87 ± 0.20
rA**G**C	10.06[12]	10.40 ± 0.2	10.23 ± 0.15
rCA**G**CA	10.44[12]	10.69 ± 0.27	10.28 ± 0.15
rC**U**C	9.47[9]	9.93 ± 0.12	9.88 ± 0.09
r**UUUUU**	10.05[9]	10.46 ± 0.07	10.55 ± 0.10

Table S12: Table of reference experimental pK_a values at 0.1M and 300 K, and final pK^{mod} values for the titratable nucleobase sites after both calibration procedures.

Nucleobases	Reference pK_a	pK^{mod} value
Adenine	3.50	3.15
Cytosine	4.22	3.90
Guanine	9.22	9.17
Uracil	9.22	8.84

Table S13: Individual pK_a values for the titratable r**UUUUU** sites. The pK_a values were obtained through a WHAM reweighting procedure using the protonation data of all CpHMD simulations. This procedure reweights each simulation's pH and protonation contributions to provide an improved estimate of the protonation averages at any arbitrary pH. The errors were determined using the bootstrap procedure for each site (N=500).

Uridine Sites	pK_a
Site1	10.08 ± 0.15
Site2	10.84 ± 0.13
Site3	11.00 ± 0.18
Site4	10.72 ± 0.14
Site5	10.28 ± 0.16

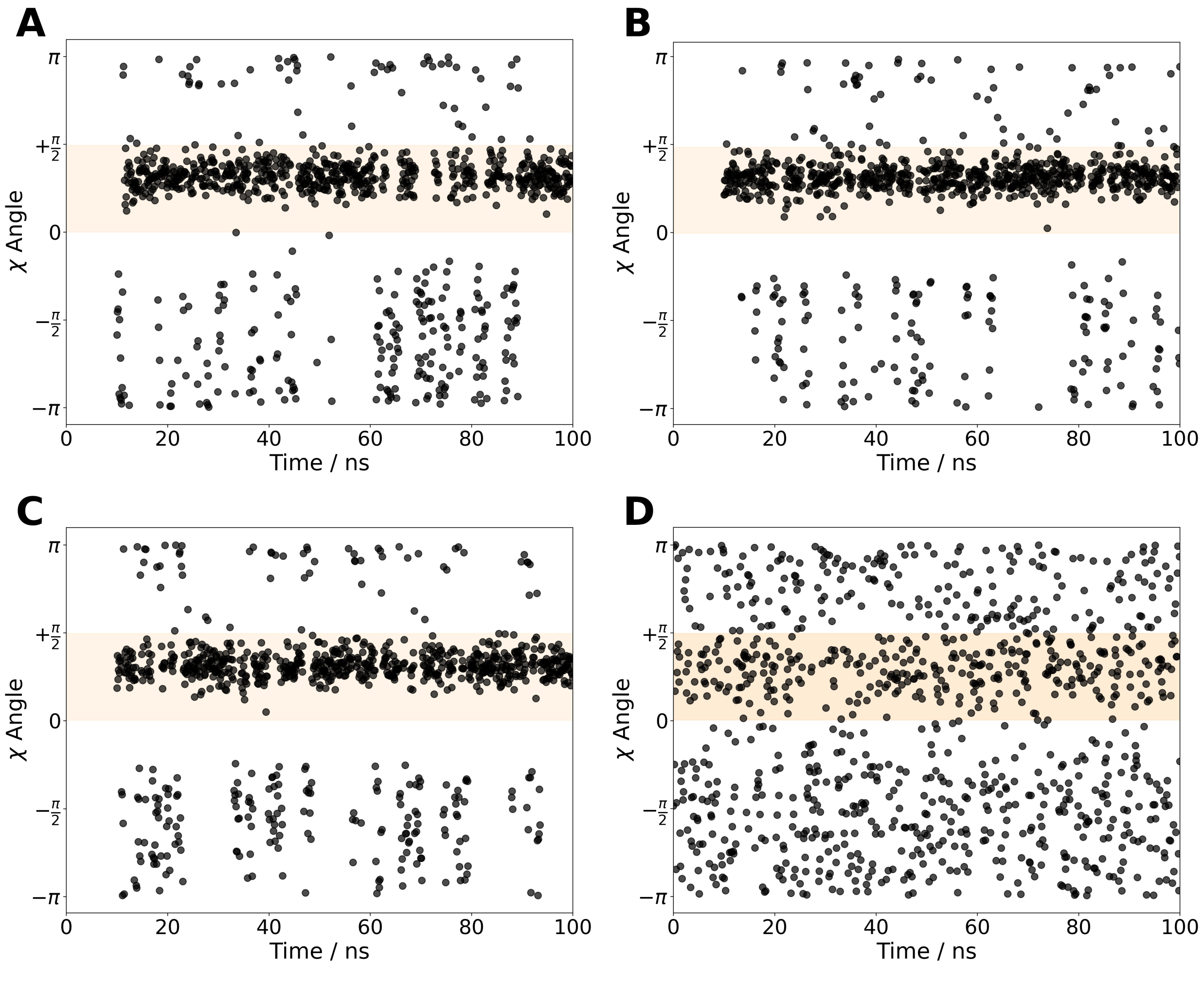

Figure S1: Timeseries of the χangle between the adenine nucleobase and the sugar. Panels **A** through **C** depict the data from different 100 ns replicates of standard st-CpHMD simulations at pH = 3.5. Panel **D** shows data from a 100 ns simulation of CpH-metaD at pH = 3.5. The green shaded regions highlight the range of values corresponding to a *syn* state.

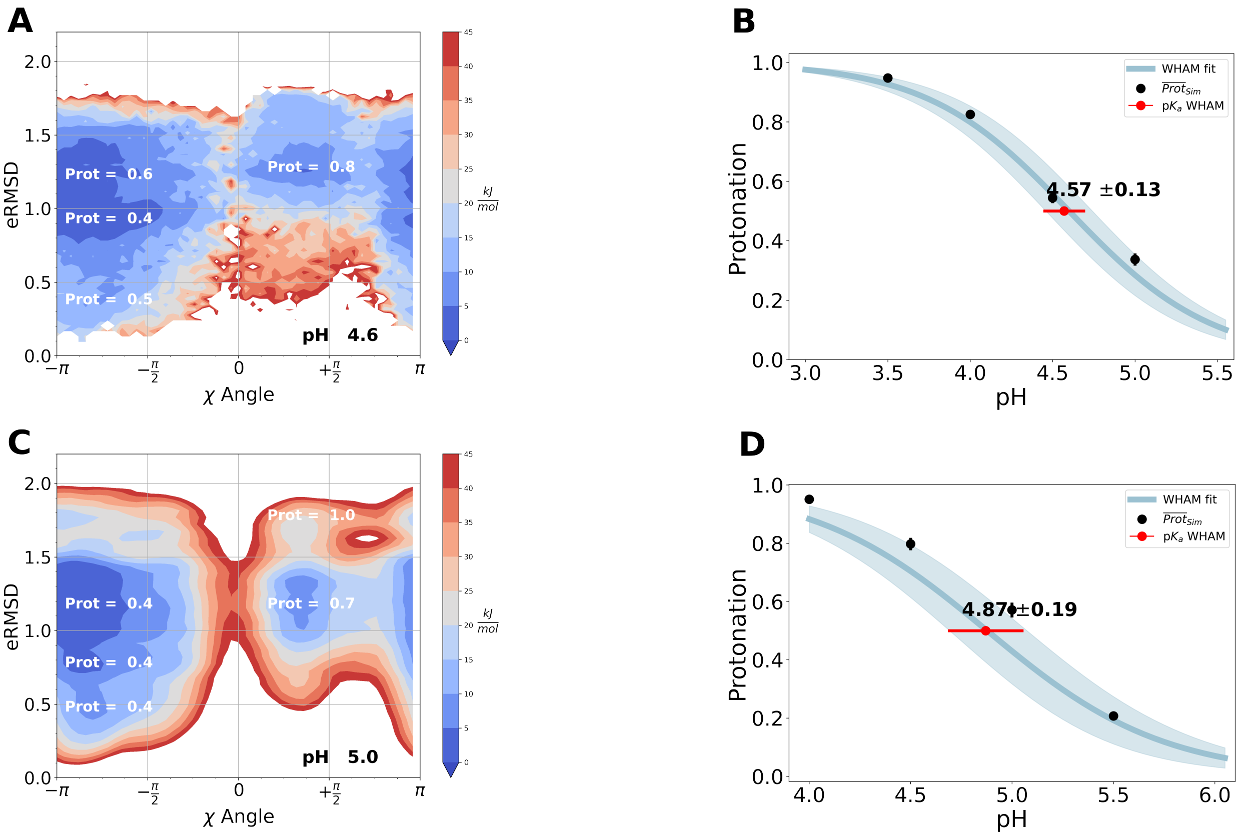

Figure S2: CpH-MetaD simulations estimate the pK_a shifts in the cytosine systems. In the left subplots, 2D energy plots display the stacking interactions (eRMSD) versus the χ angle of the titratable adenine. The major energy minima are identified by their average protonation (i.e., the population of protonated conformations) and are labeled as "Prot". In the right subplots, the pK_a values (red circles) and titration curves (gray lines) for rU**C**U/rUU**C**UU were obtained through a WHAM reweighting procedure using equilibrated data from all CpH simulations. Each simulation's average protonation and respective errors are plotted as black circles. The standard errors were estimated using a bootstrap method.

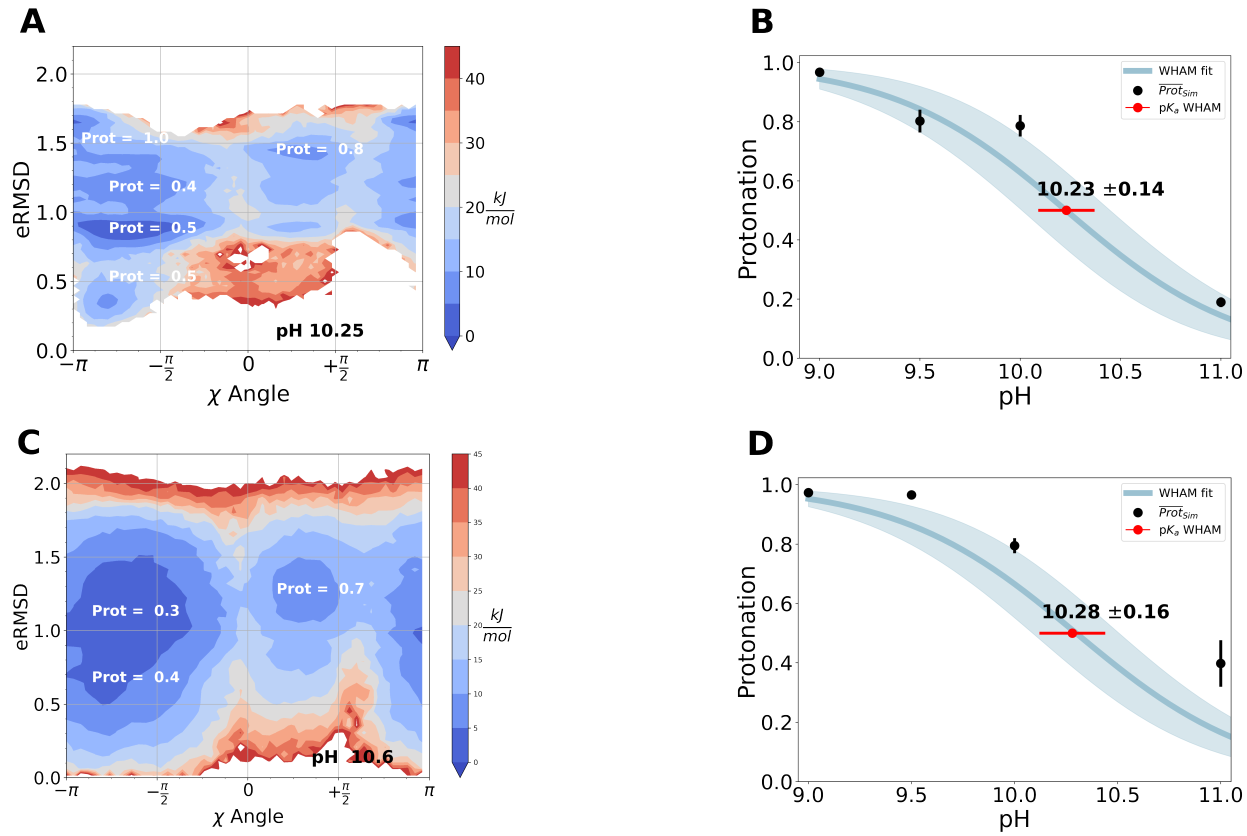

Figure S3: CpH-MetaD simulations estimate the pK_a shifts in the guanosine systems. In the left subplots, 2D energy plots display the stacking interactions (eRMSD) versus the χ angle of the titratable guanosine. The major energy minima are identified by their average protonation (i.e., population of protonated conformations) and are labeled as "Prot". In the right subplots, the pK_a values (red circles) and titration curves (gray lines) for rA**G**C/rCA**G**CA were obtained through a WHAM reweighting procedure using equilibrated data from all CpH simulations. Each simulation's average protonation and respective errors are plotted as black circles. The standard errors were estimated using a bootstrap method.

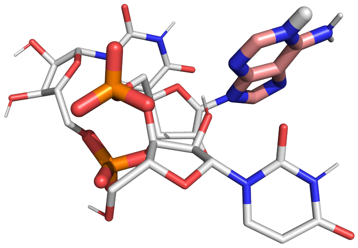

(a) Cartoon representation of a representative syn state for rU**A**U.

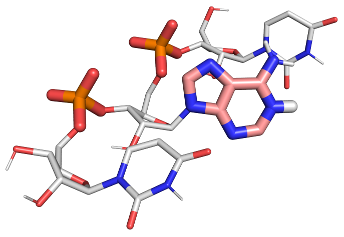

(b) Network Cartoon representation of a representative low eRMSD state for rU**A**U.

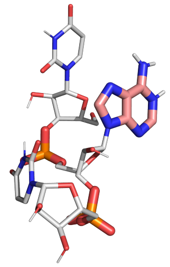

(c) Cartoon representation of a representative high eRMSD state for rU**A**U.

Figure S4: Backbone carbons are shown in white, while the titratable group carbons are depicted in gray. Nitrogen atoms are represented in dark blue and oxygen and phosphorous atoms are displayed as red and orange sticks, respectively. The centroid frame of each energy minimum is derived from the 2D energy maps shown in Figure S16.

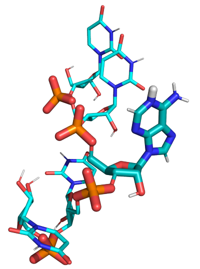

(a) Cartoon representation of a representative syn state for rUU**A**UU.

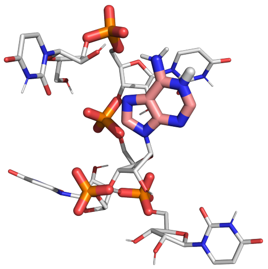

(b) Cartoon representation of a representative low eRMSD state for rUU**A**UU.

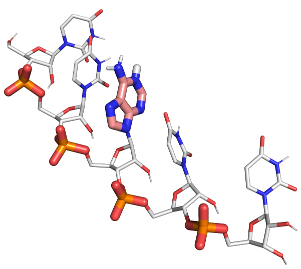

(c) Cartoon representation of a representative medium eRMSD state for rUU**A**UU.

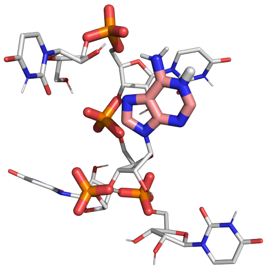

(d) Cartoon representation of a representative high eRMSD state for rUU**A**UU.

Figure S5: Backbone carbons are shown in white, while the titratable group carbons are depicted in gray. Nitrogen atoms are represented in dark blue and oxygen and phosphorous atoms are displayed as red and orange sticks, respectively. The centroid frame of each energy minimum is derived from the 2D energy maps shown in Figure S11.

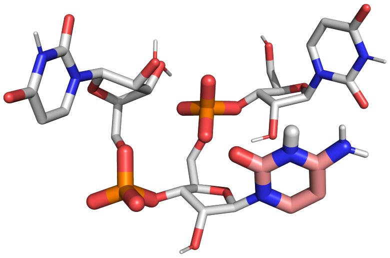

(a) Cartoon representation of a representative syn state for rU**C**U.

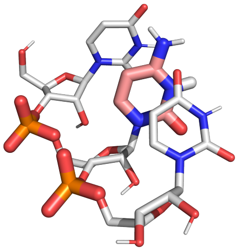

(b) Network Cartoon representation of a representative low eRMSD state for rU**C**U.

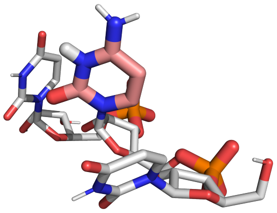

(c) Cartoon representation of a representative medium eRMSD state for rU**C**U.

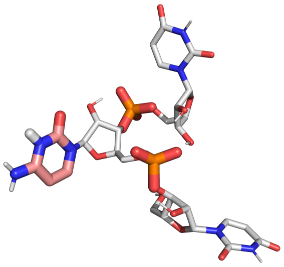

(d) Cartoon representation of a representative high eRMSD state for rU**C**U.

Figure S6: Backbone carbons are shown in white, while the titratable group carbons are depicted in gray. Nitrogen atoms are represented in dark blue, and oxygen and phosphorous atoms are displayed as red and orange sticks, respectively. The centroid frame of each energy minimum is derived from the 2D energy maps shown in Figure S13

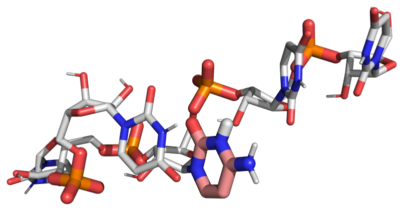

(a) Cartoon representation of a representative syn state for rUU**C**UU.

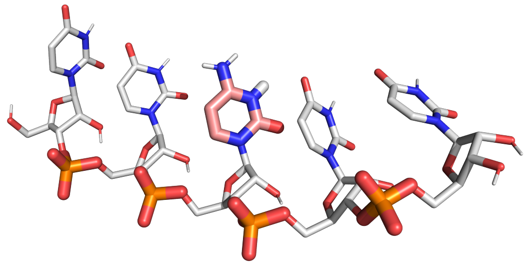

(b) Cartoon representation of a representative low eRMSD state for rUU**C**UU.

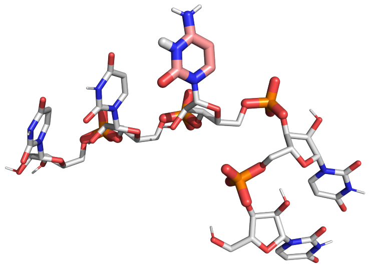

(c) Cartoon representation of a representative medium eRMSD state for rUU**C**UU.

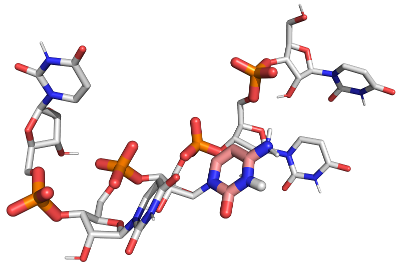

(d) Cartoon representation of a representative high eRMSD state for rUU**C**UU.

Figure S7: Backbone carbons are shown in white, while the titratable group carbons are depicted in gray. Nitrogen atoms are represented as dark blue, and oxygen and phosphorous atoms are displayed as red and orange sticks, respectively. The centroid frame of each energy minimum is derived from the 2D energy maps shown in Figure S14.

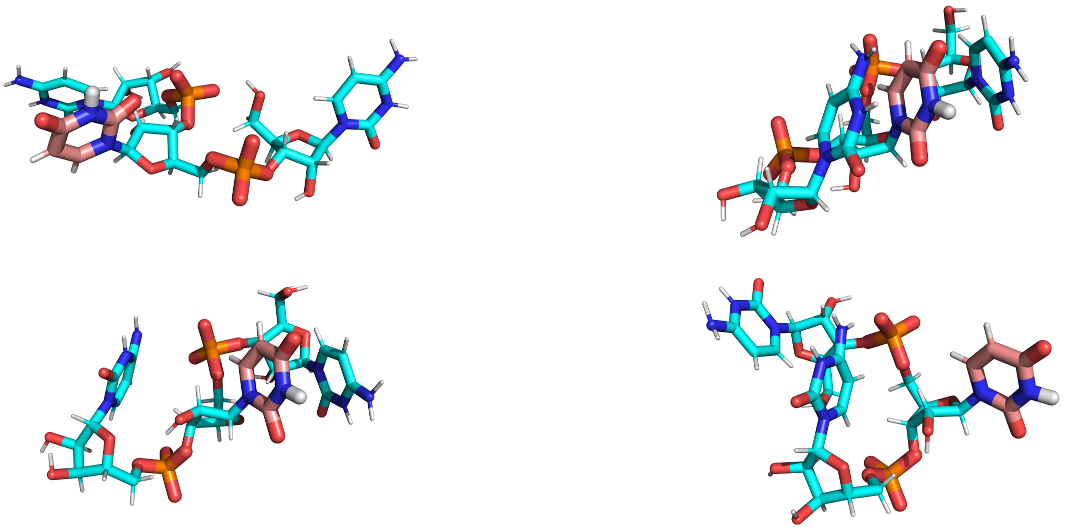

Figure S8

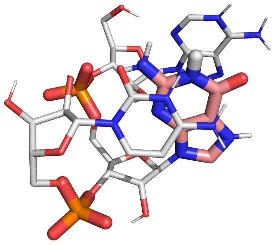

(a) Cartoon representation of a representative syn state for rA**G**C.

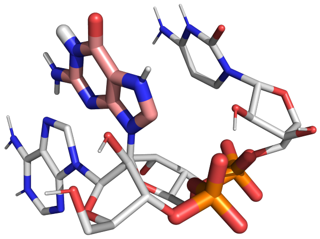

(b) Network Cartoon representation of a representative low eRMSD state for rA**G**C.

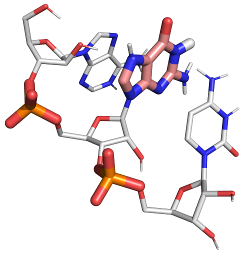

(c) Cartoon representation of a representative medium low eRMSD state for rA**G**C.

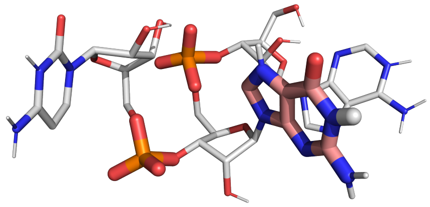

(d) Cartoon representation of a representative medium high eRMSD state for rA**G**C.

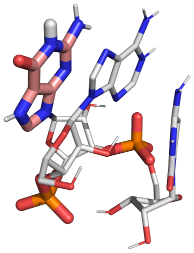

(e) Cartoon representation of a representative high eRMSD state for rA**G**C.

Figure S9: Backbone carbons are shown in white, while the titratable group carbons are depicted in gray. Nitrogen atoms are represented as dark blue, and oxygen and phosphorous atoms are displayed as red and orange sticks, respectively. The centroid frame of each energy minimum is derived from the 2D energy maps shown in Figure S18

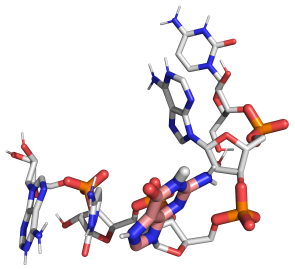

(a) Cartoon representation of a representative syn state for rCA**G**CA.

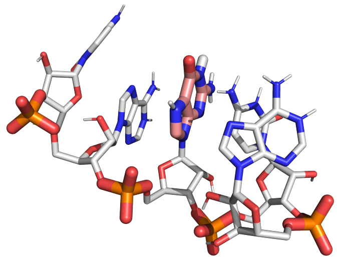

(b) Network Cartoon representation of a representative low eRMSD state for rCA**G**CA.

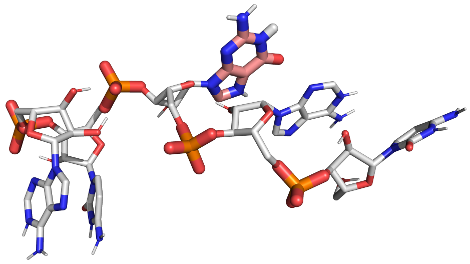

(c) Cartoon representation of a representative medium eRMSD state for rCA**G**CA.

Figure S10: Backbone carbons are shown in white, while the titratable group carbons are depicted in gray. Nitrogen atoms are represented in dark blue, and oxygen and phosphorous atoms are displayed as red and orange sticks, respectively. The centroid frame of each energy minimum is derived from the 2D energy maps shown in Figure S20

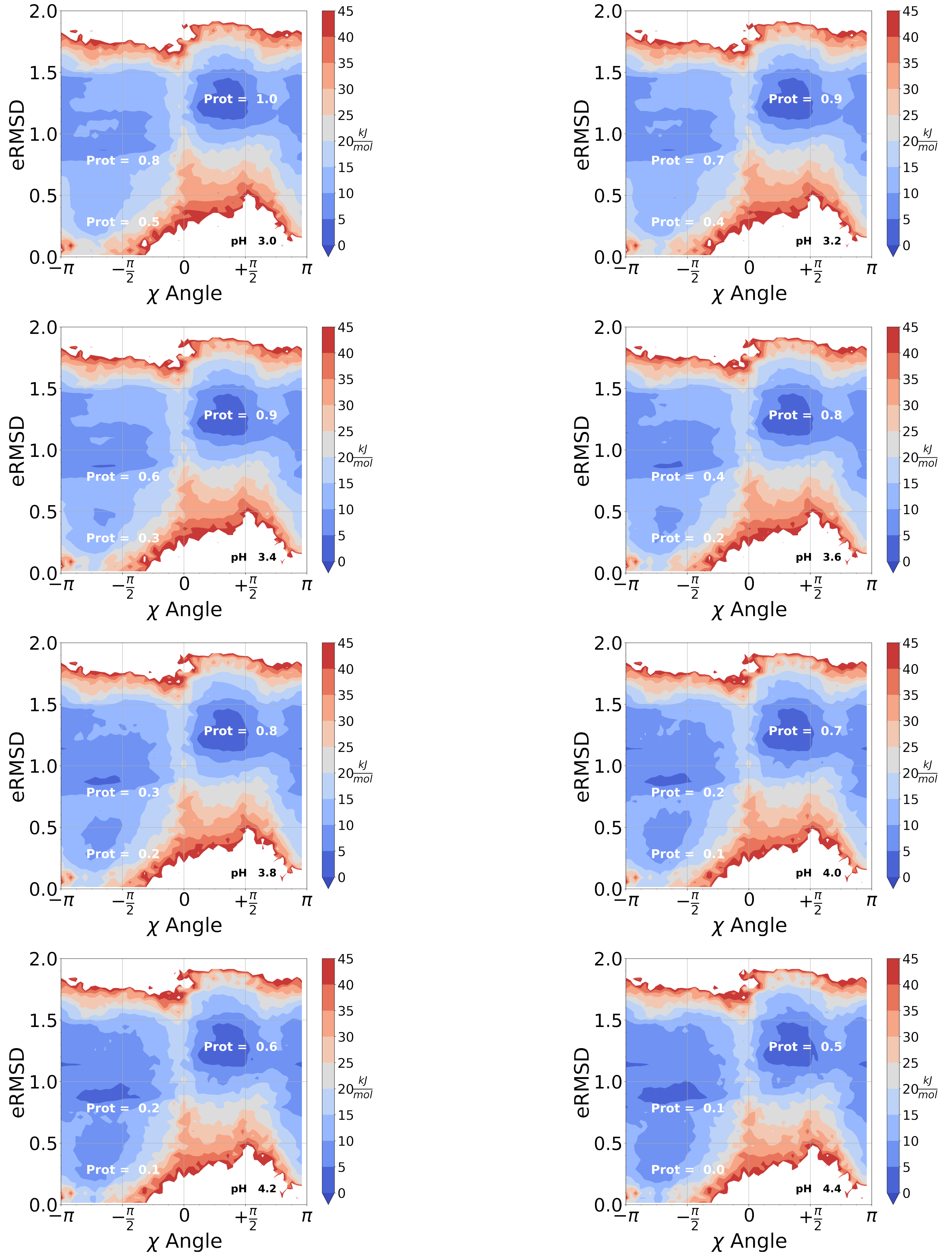

Figure S11: 2D energy maps along the chosen collective variables- the χ angle of protonable adenine and the eRMSD relative to a fully stacked rU**A**U oligomer. Each energy minimum is identified by the average protonation computed over all values of the pH range of 3.0 to 4.4.

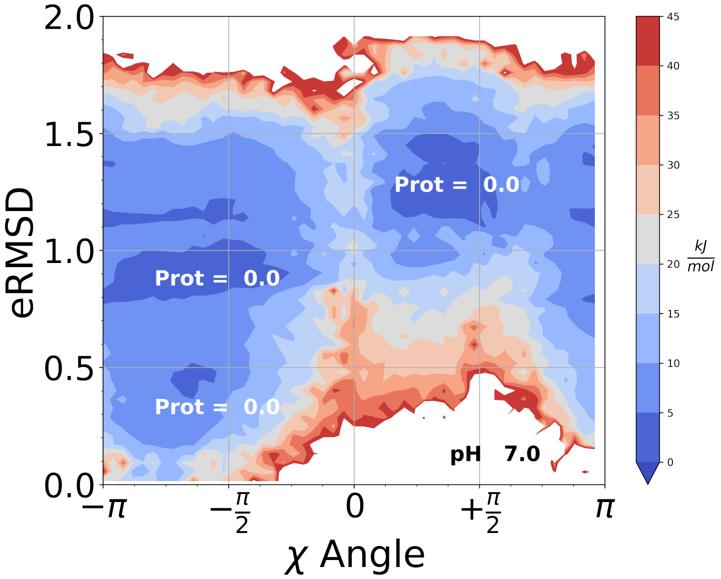

Figure S12: 2D energy maps along the chosen collective variables- the χ angle of protonable adenine and eRMSD relative to a fully stacked rU**A**U oligomer at physiological pH conditions. Each energy minimum is identified by the average protonation.

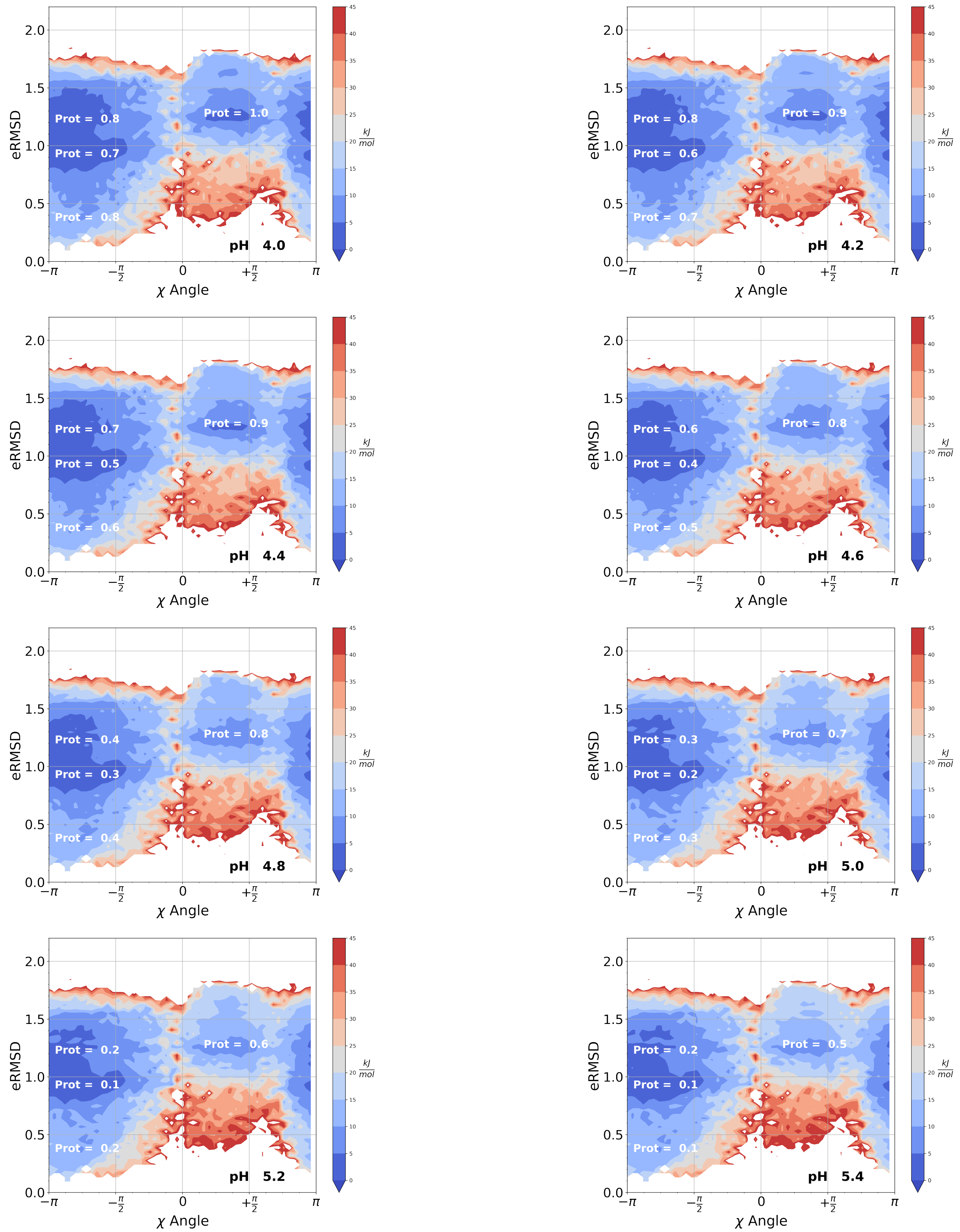

Figure S13: 2D energy maps along the chosen collective variables- the χ angle of protonable cytosine and the eRMSD relative to a fully stacked rU**C**U oligomer. Each energy minimum is identified by the average protonation computed over all values of the pH range of 4.0 to 5.4.

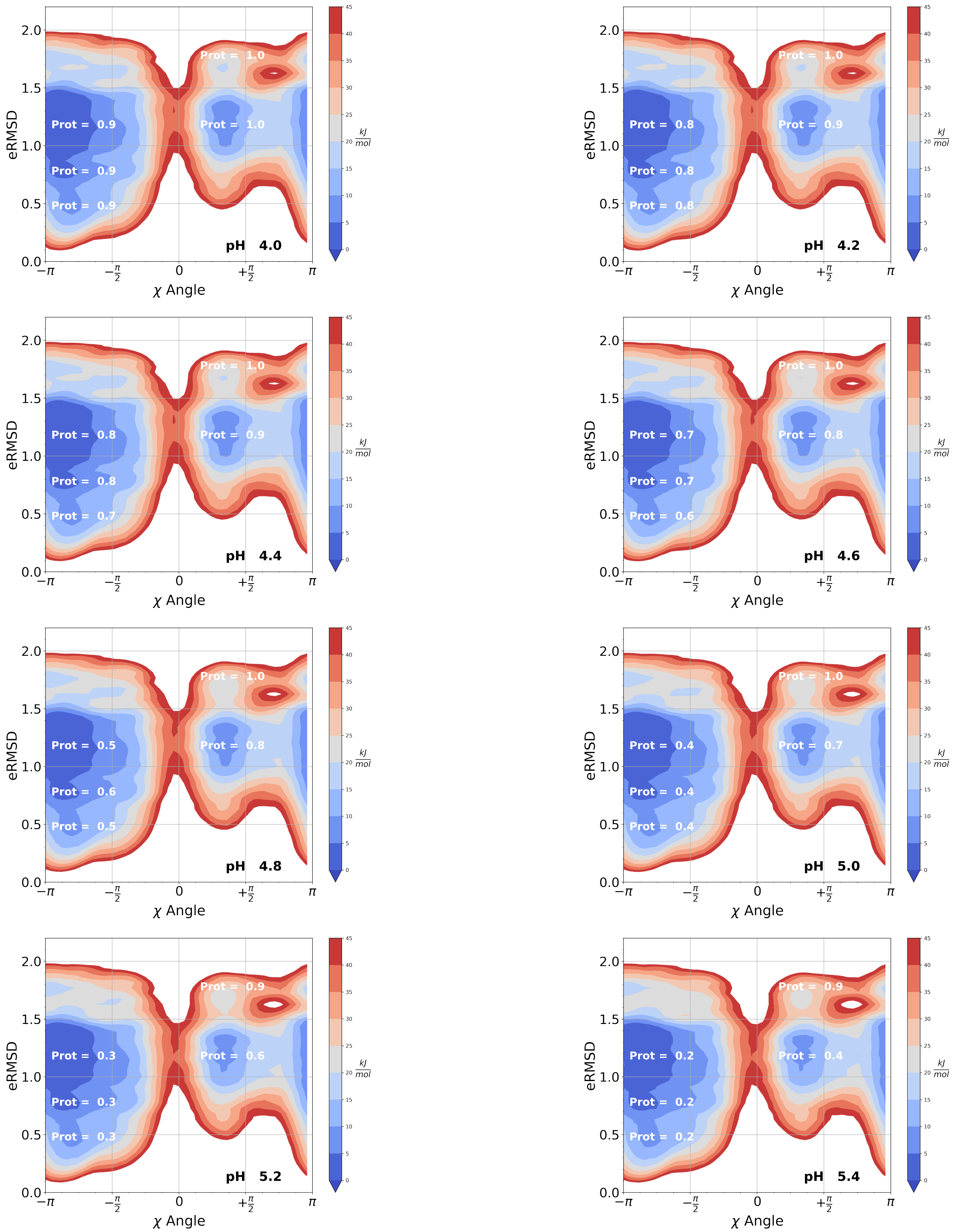

Figure S14: 2D energy maps along the chosen collective variables- the χ angle of protonable cytosine and the eRMSD relative to a fully stacked rUU**C**UU oligomer. Each energy minimum is identified by the average protonation computed over all values of the pH range of 4.0 to 5.4.

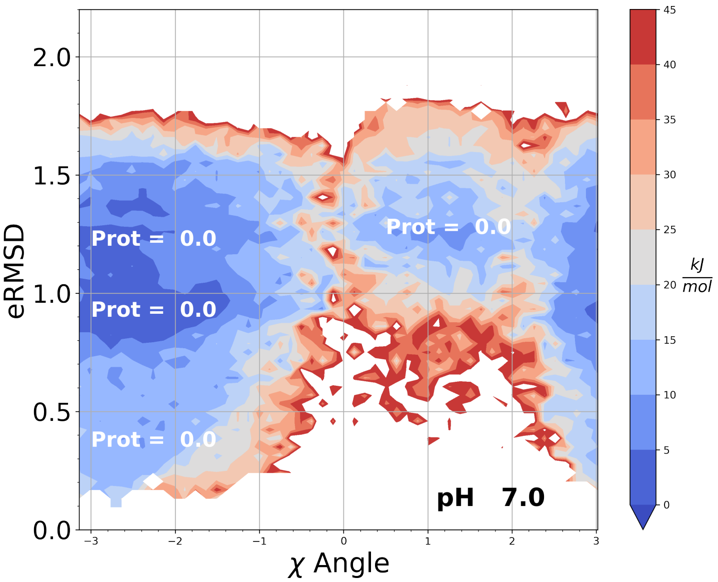

Figure S15: 2D energy maps along the chosen collective variables- the χ angle of protonable cytosine and the eRMSD relative to a fully stacked rU**A**U oligomer at physiological pH conditions. Each energy minimum is identified by the average protonation.

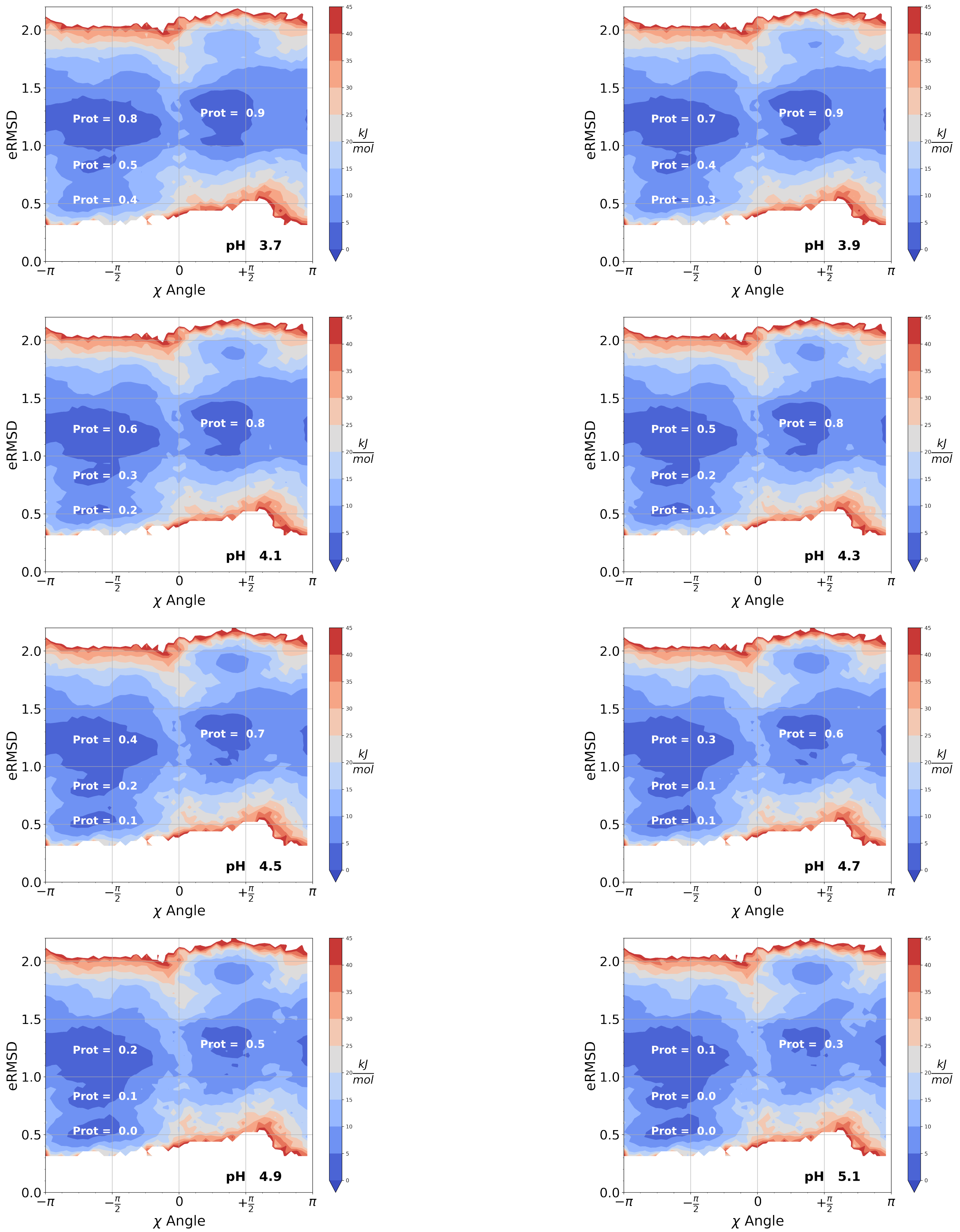

Figure S16: 2D energy maps along the chosen collective variables- the χ angle of titratable adenine and the eRMSD relative to a fully stacked rUU**A**UU oligomer. Each energy minimum is identified by the average protonation computed over all values of the pH range of 3.7 to 5.1.

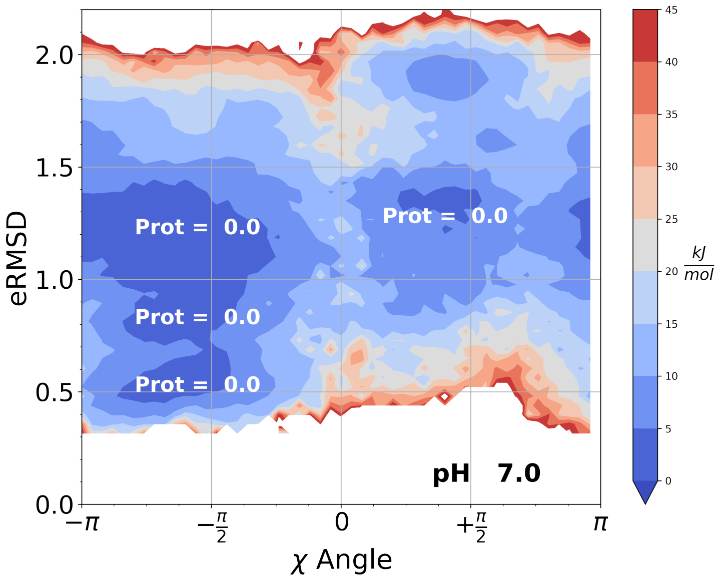

Figure S17: 2D energy maps along the chosen collective variables- the χ angle of protonable adenine and the eRMSD relative to a fully stacked rUU**A**UU oligomer at physiological pH value. Each energy minimum is identified by the average protonation.

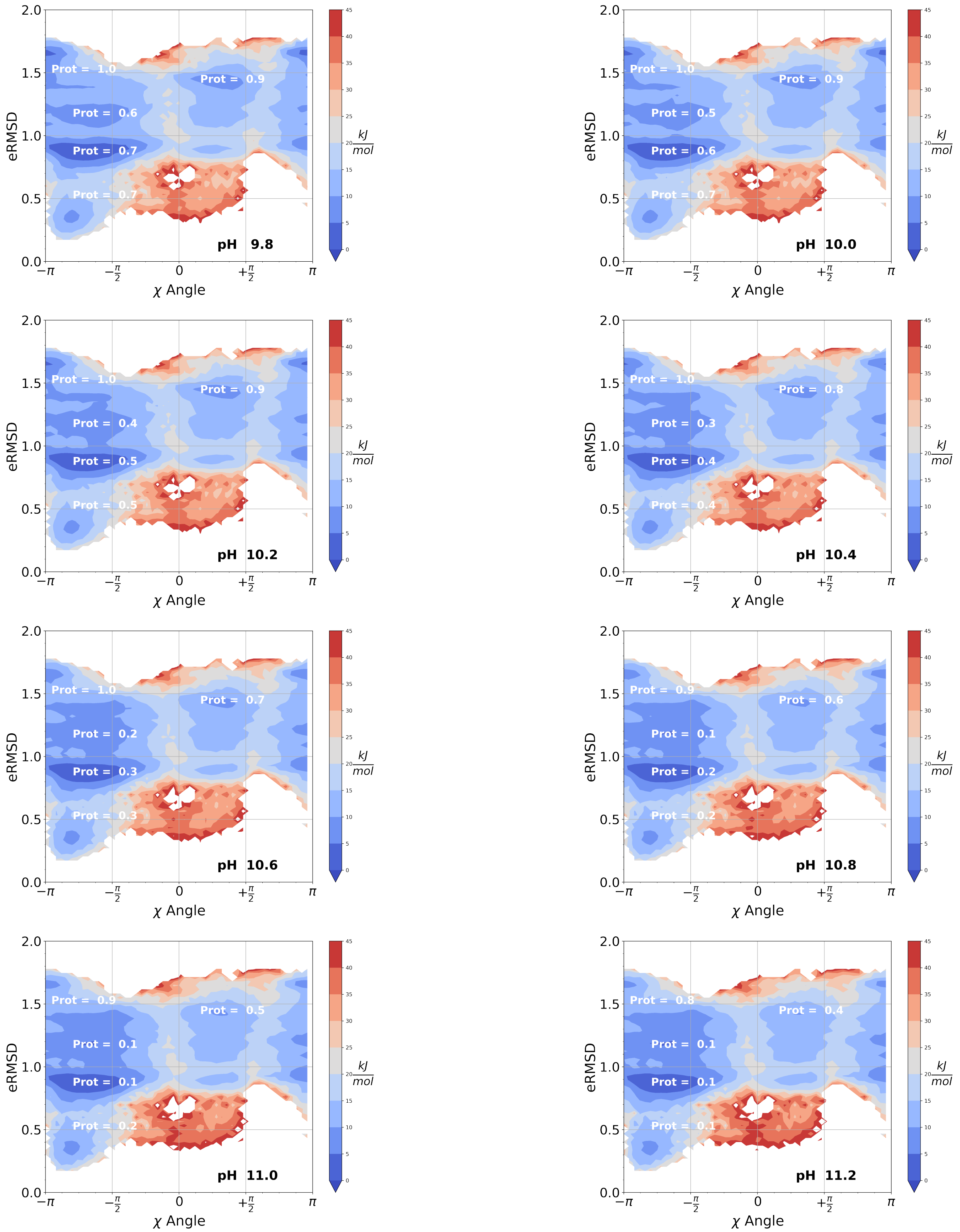

Figure S18: 2D energy maps along the chosen collective variables- the χ angle of deprotonable guanine and the eRMSD relative to a fully stacked rA**G**C oligomer. Each energy minimum is identified by the average protonation computed over all values of the pH range of 9.8 to 11.2.

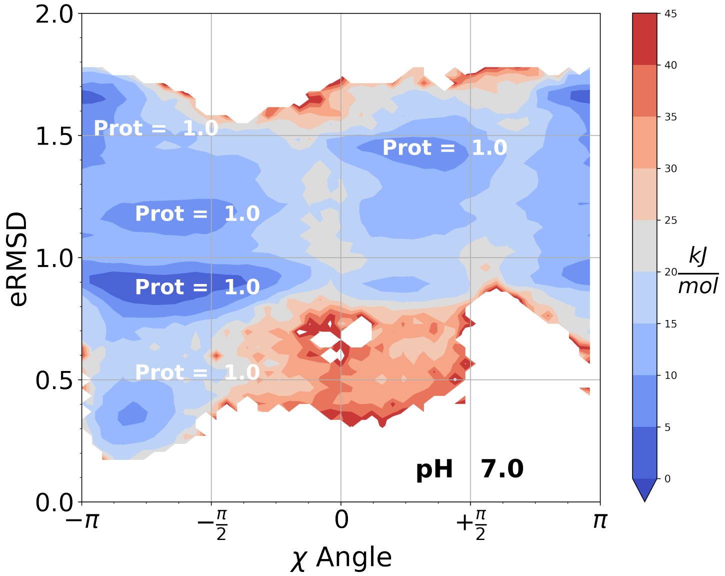

Figure S19: 2D energy maps along the chosen collective variables- the χ angle of deprotonable guanine and the eRMSD relative to a fully stacked rA**G**C oligomer at a physiological pH value. Each energy minimum is identified by the average protonation.

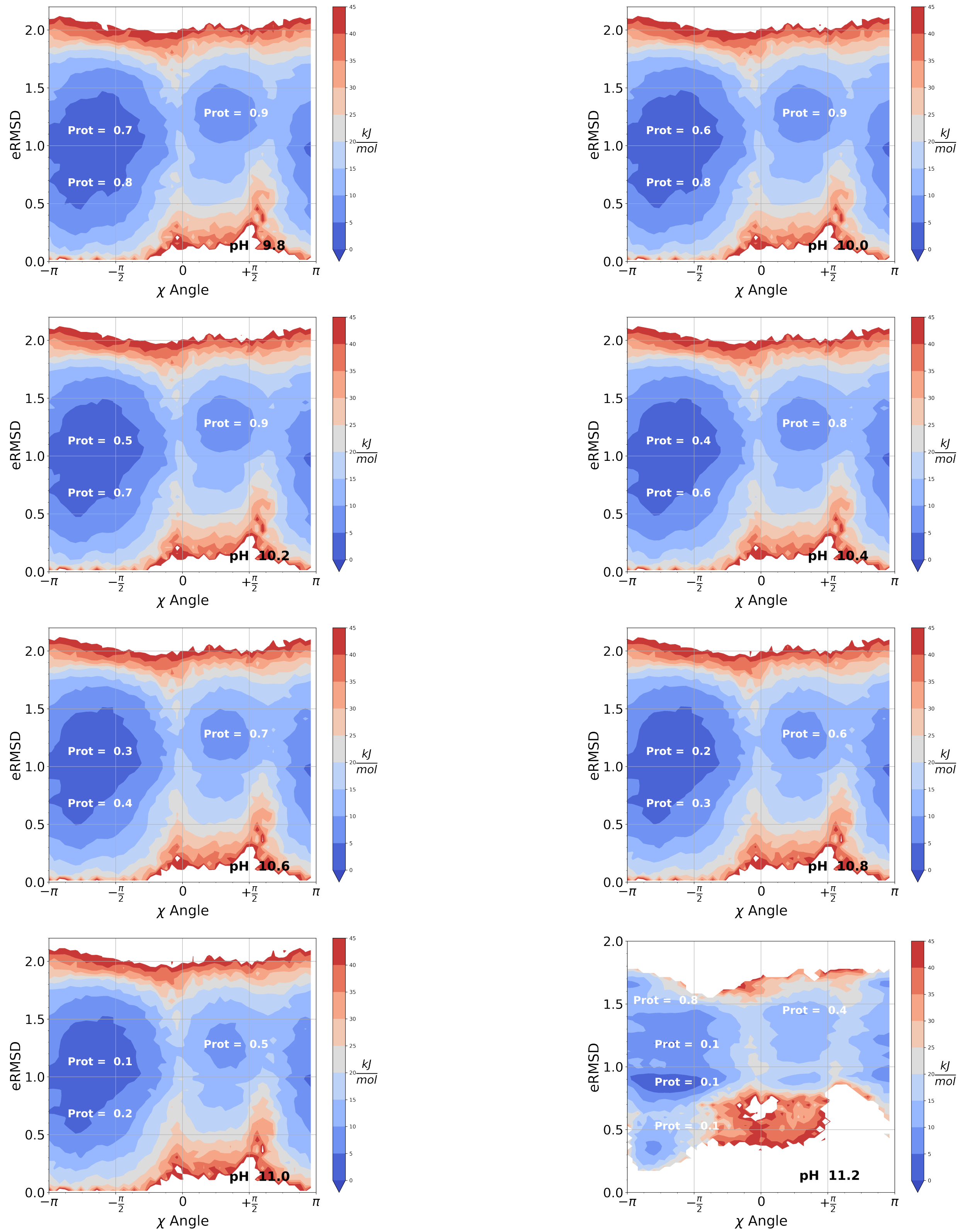

Figure S20: 2D energy maps along the chosen collective variables- the χ angle of deprotonable guanine and the eRMSD relative to a fully stacked rCA**G**CA oligomer. Each energy minimum is identified by the average protonation computed over all values of the pH range of 9.8 to 11.2.

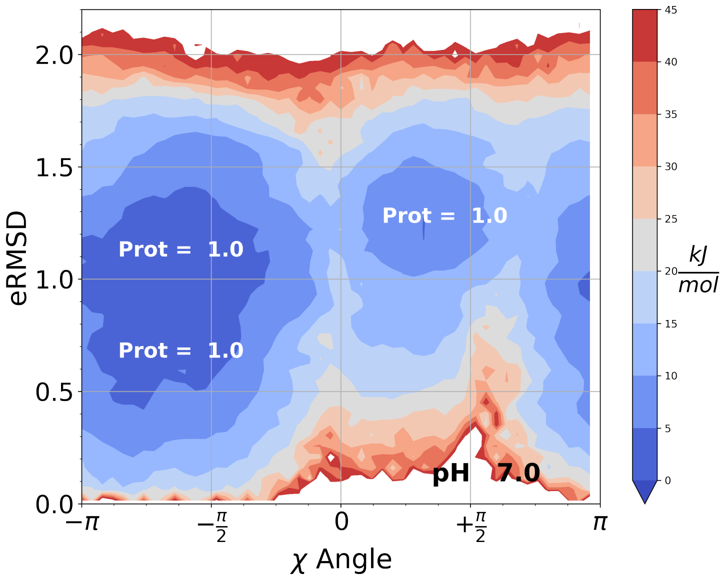

Figure S21: 2D energy maps along the chosen collective variables- the χ angle of deprotonable guanine and the eRMSD relative to a fully stacked rCA**G**CA oligomer at a physiological pH value. Each energy minimum is identified by the average protonation.

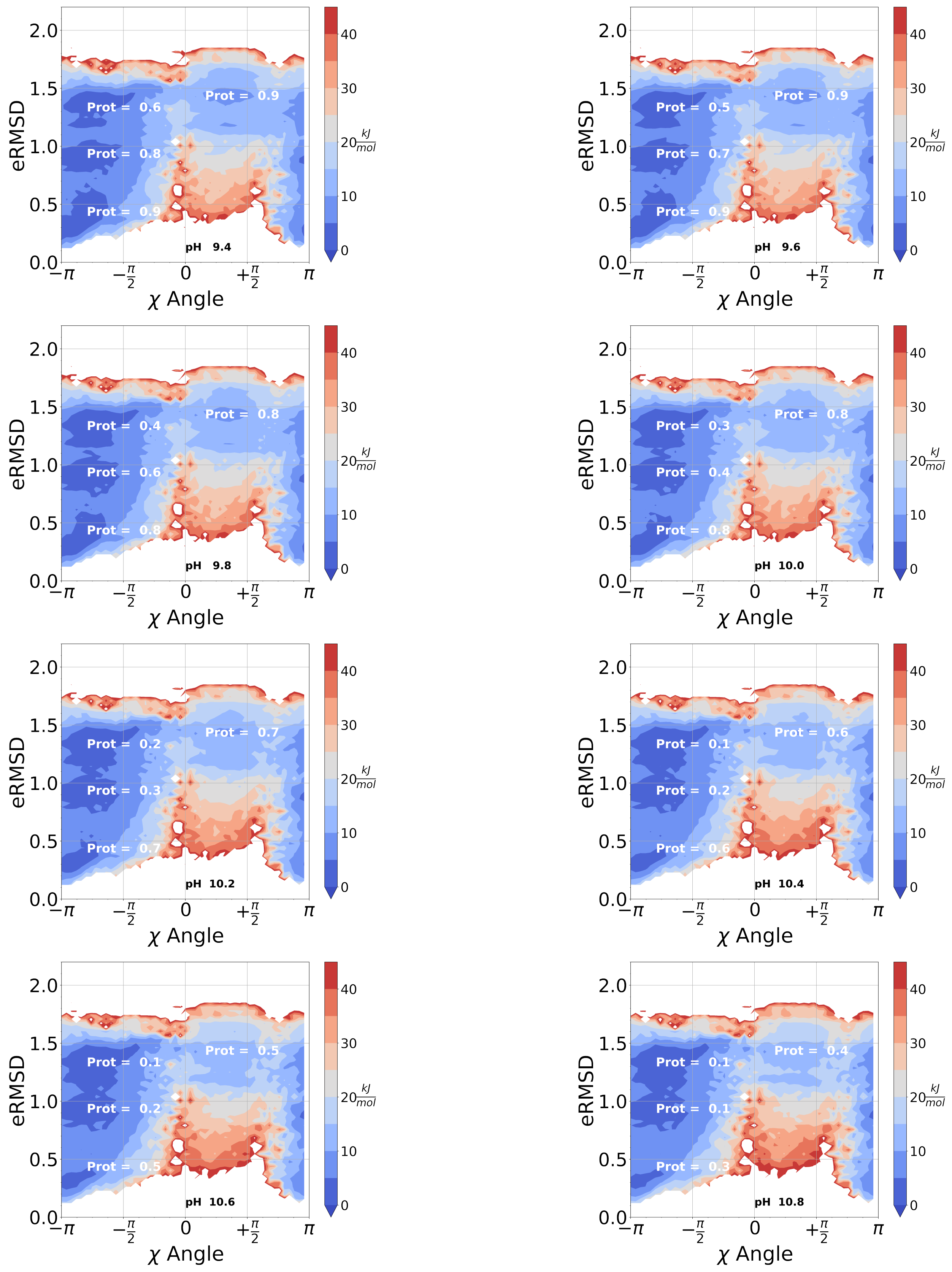

Figure S22: 2D energy maps along the chosen collective variables- the χ angle of deprotonable uridine and the eRMSD relative to a fully stacked rC**U**C oligomer. Each energy minimum is identified by the average protonation computed over all values of the pH range of 9.4 to 10.8.

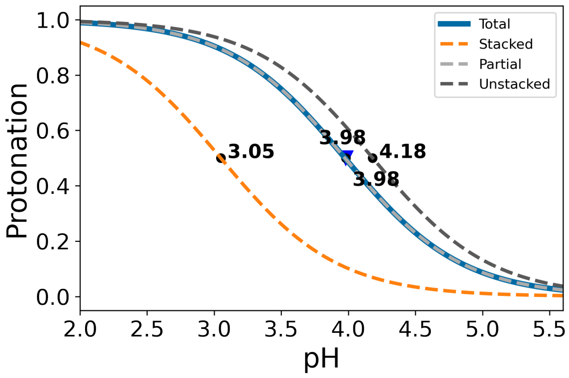

(a) rU**A**U

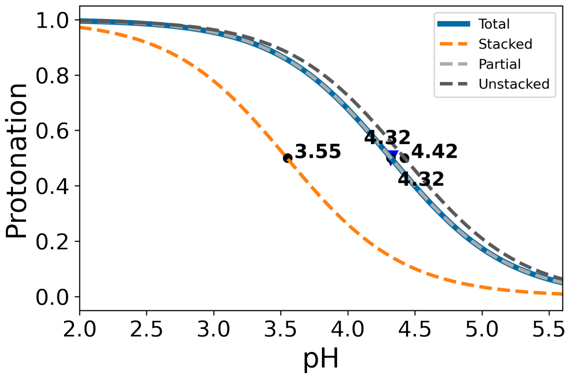

(b) rUU**A**UU

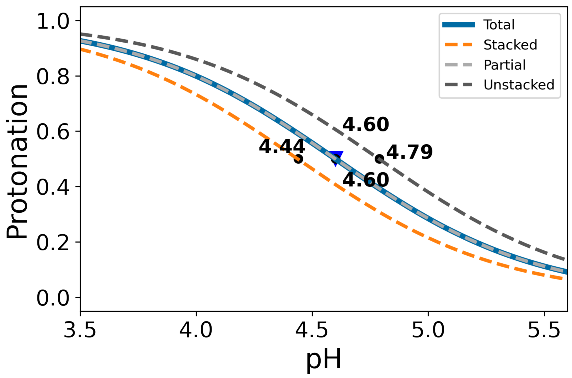

(c) rU**C**U

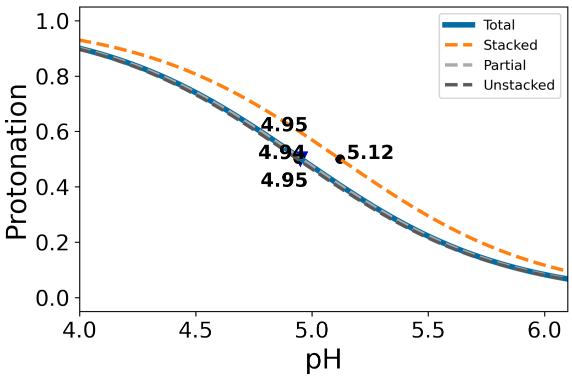

(d) rUU**C**UU

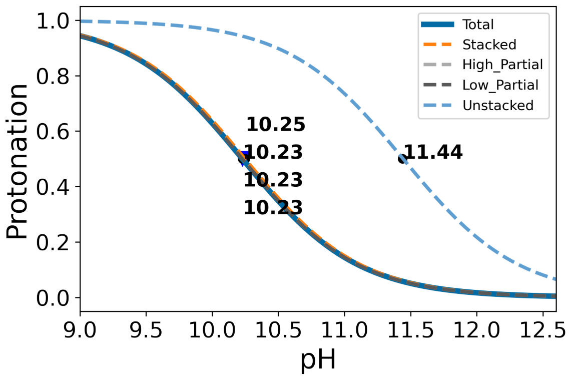

(e) rA**G**C

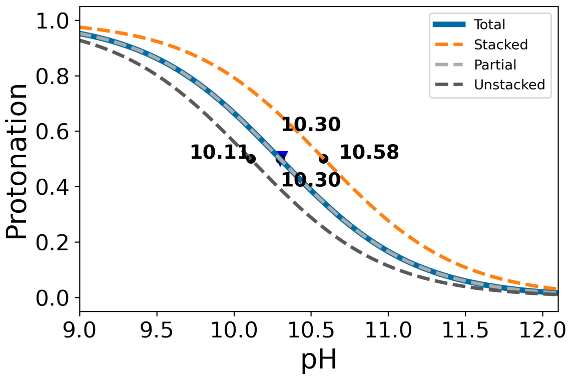

(f) rCA**G**CA

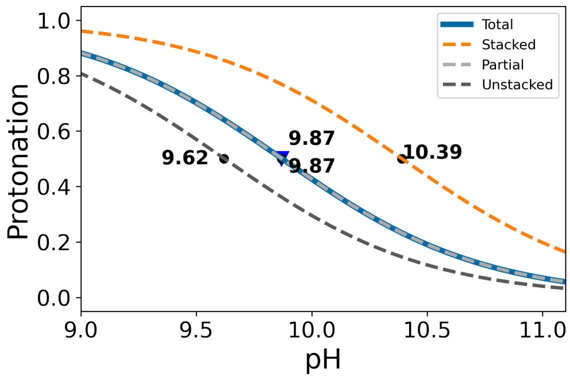

(g) rC**U**C

Figure S23: Base-stacking-dependent titration curves and pK_a values for all studied systems, except for the r**UUUUU** system. The total curve represents the WHAM fit using all available data. The plotted data were obtained using the binless WHAM procedure.

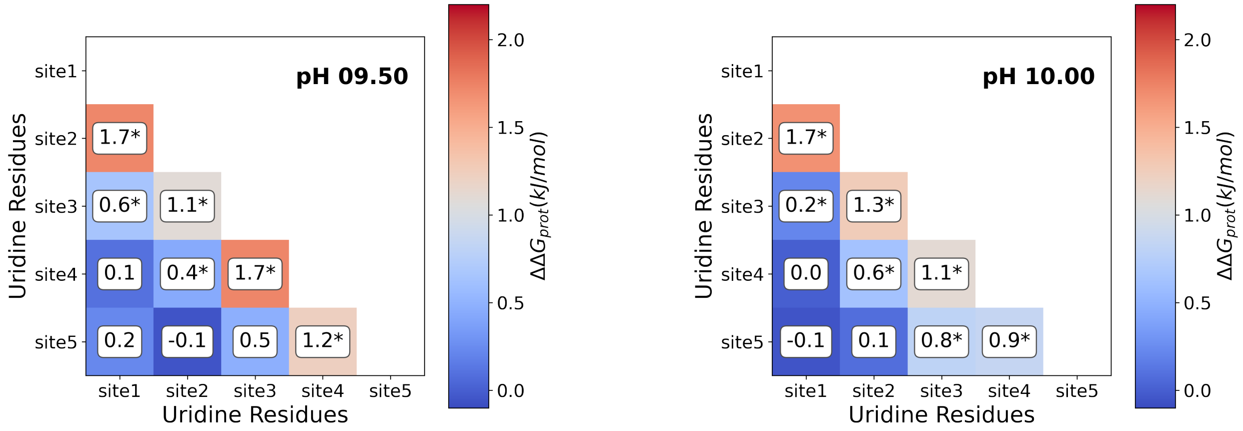

Figure S24: Pairwise protonation cooperativity matrix for r**UUUUU** at pH 9.5 and 10. The ΔΔG values indicate the likelihood of simultaneous protonation of two sites in a multi-titratable strand, with positive values signifying a higher barrier. Statistical significance (*) was determined using the Benjamini-Hochberg method[86] following a bootstrapping procedure (n=1000).
